# Screw dislocation-induced pyramidal crystallization of dendron-like macromolecules featuring asymmetric geometry†

**DOI:** 10.1039/d1sc02617h

**Published:** 2021-08-06

**Authors:** Xinyu Sun, Xueyan Feng, Xiao-Yun Yan, Jiancheng Luo, Ruimeng Zhang, Tao Li, Hui Li, Jiahui Chen, Fangbei Liu, Ehsan Raee, Stephen Z. D. Cheng, Tianbo Liu

**Affiliations:** School of Polymer Science and Polymer Engineering, The University of Akron Akron Ohio 44325 USA tliu@uakron.edu scheng@uakron.edu; South China Advanced Institute for Soft Matter Science and Technology, School of Molecular Science and Engineering, South China University of Technology Guangzhou 510640 China; X-Ray Science Division, Advanced Photon Source, Argonne National Laboratory Argonne IL 60439 USA; Department of Chemistry and Biochemistry, Northern Illinois University DeKalb IL 60115 USA

## Abstract

We report herein that dendron-shaped macromolecules AB_*n*_ crystallize into well-ordered pyramid-like structures from mixed solvents, instead of spherical motifs with curved structures, as found in the bulk. The design of the asymmetric molecular architecture and the choice of mixed solvents are applied as strategies to manipulate the crystallization process. In mixed solvents, the solvent selection for the Janus macromolecule and the existence of dominant crystalline clusters contribute to the formation of flat nanosheets. Whereas during solvent evaporation, the bulkiness of the asymmetric macromolecules easily creates defects within 2D nanosheets which lead to their spiral growth through screw dislocation. The size of the nanosheets and the growth into 2D nanosheets or 3D pyramidal structures can be regulated by the solvent ratio and solvent compositions. Moreover, macromolecules of higher asymmetry generate polycrystals of lower orderliness, probably due to higher localized stress.

## Abstract 2

The dendron-shaped macromolecules AB*_n_* crystallize into well-ordered pyramid-like structures from mixed solvents, which is on the contrary to spherical motifs with curved structures in bulk.

## Graphical abstract

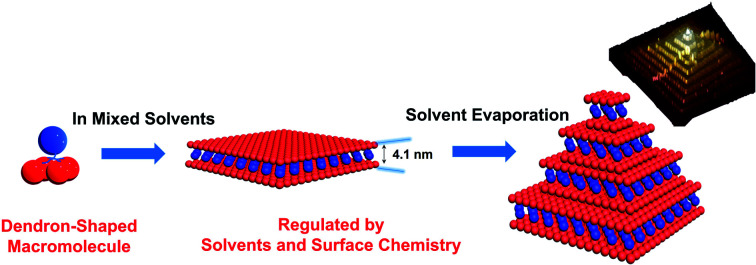

## Introduction

During the crystallization process, the crystal morphology is often affected by sets of robust intermolecular interactions.^1–3^ In order to control the crystal phase and materials properties, various crystal engineering strategies are applied such as structure modification and regulation of molecular concentration, orientation, and nucleation rate through solution and vapor processing methodologies.^4–8^ Under the influence of stress or impurities, real crystals always have structural disorder which introduces heterogeneity without losing the long-range order. As the atom(s) are located at irregular positions, many interesting and unexpected supramolecular architectures were observed. For example, materials undergoing spiral growth driven by screw dislocation were constructed into 1D nanotubes,^9,10^ 1D nanowires,^11–13^ and 2D nanoplates.^14–16^ Crystal growth through screw dislocation was discovered and studied in the crystallization of inorganic materials,^10,15–19^ organic molecules,^20,21^ polymers,^22–25^ and even viruses,^26^ especially through the solution and vapor-grown methods. However, the screw dislocation-driven crystal growth of macromolecules has not been reported yet.

In the past decade, studies on self-assembled hierarchical structures have been extended from small molecules to macromolecules, among which the molecular nanoparticles (MNPs) are promising representatives, such as polyhedral oligomeric silsesquioxanes (POSS), polyoxometalates (POMs), and fullerene (C_60_).^27–32^ Tethering the MNPs with organic components or different types of MNPs through chemical modification can build up MNP-based giant surfactants and molecular Janus particles (MJPs). Their characteristic anisotropy in both topology and chemistry can be designed to meet the needs of various functions, amphiphilicity, macromolecular shapes, and sequences.^33–37,40–44,77,78^ Due to the anisotropy as well as the rigid domains, they have enriched the phase diagrams by self-assembling into diverse nanostructures in the bulk, at the interface, or in solutions.^38,39,45–55^

The topological design featured by breaking the geometrical symmetry of MJPs has elucidated its influence on phase behaviors.^56,57^ When increasing the degree of asymmetry, continuous changes in crystalline and quasicrystalline phases were achieved in the bulk, indicating the influence of domain volume fractions on the interface curvatures.^48,49,58–60^ For example, “Frank–Kasper” (F–K) phase structures such as A15 and sigma phases, featured by spherical motifs, were fabricated when the MJP topology deviated from symmetry to tetrahedron or dendron-like shapes.^48,49,61^ A 2D honeycomb superlattice was observed to originate from the fan-shaped geometry of the POM-4POSS macromolecule.^62^ However, there were still limited solution studies on these interesting highly asymmetric MJPs.^51^ It is expected that MJPs with a huge anisotropic topology will behave differently in solution, which can fulfill the phase diagram as well as enhance the understanding of supramolecular constructions.

**Scheme 1:**
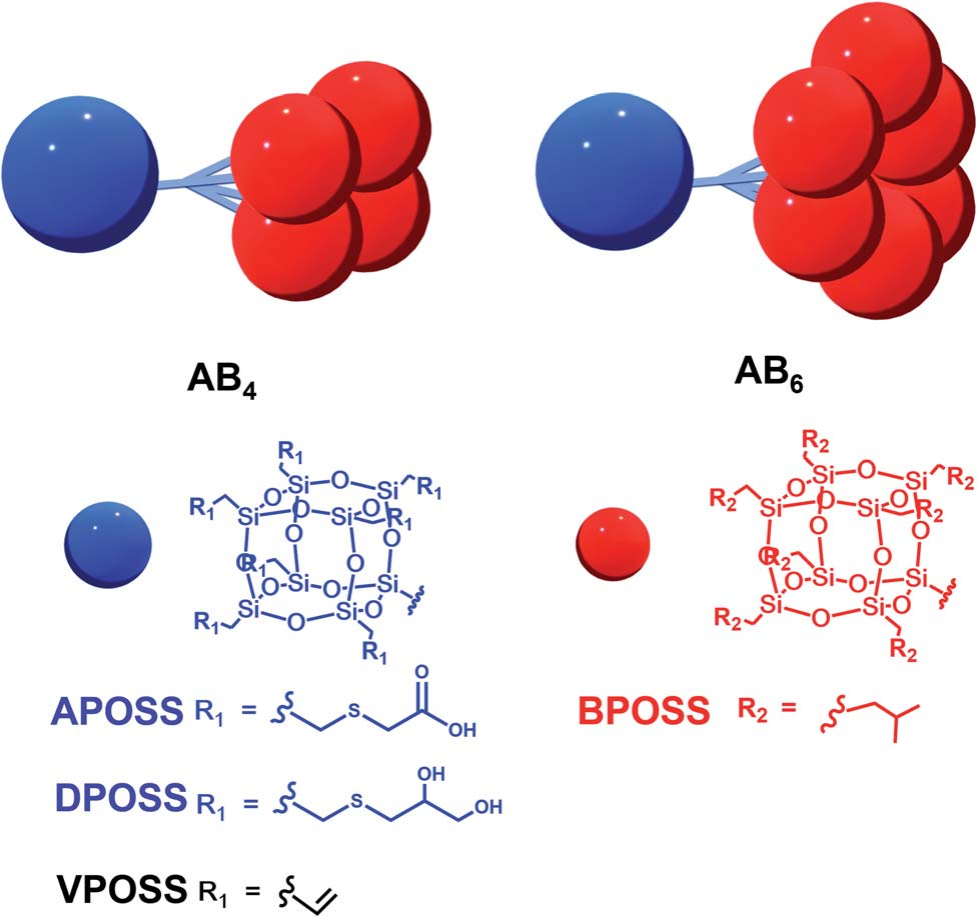
Cartoon illustrations and chemical structures of dendron-like macromolecules AB_*n*_ with different functional groups.

Herein, we used the dendron-like macromolecule AB_*n*_ (*n* = 4 or 6 in this work) as a model to demonstrate the assembly behaviors and morphologies influenced by the degree of asymmetry, surface chemistry, and choice of solvents. The AB_*n*_ structure is composed of one hydrophilic APOSS (functionalized with seven carboxyl groups) or DPOSS (functionalized with 14 hydroxyl groups) and *n* hydrophobic BPOSS (functionalized with 7 isobutyl groups) cages. The design and synthesis of AB_*n*_ macromolecules (APOSS–BPOSS_4_, DPOSS–BPOSS_4_, and APOSS–BPOSS_6_) followed the route reported before by tethering multi POSS cages *via* the dendron-type linkers, while the surface functionality was modified to meet different needs through the “thiol–ene” reaction (Scheme 1 and Fig. S1–3†).^49,63^ It is known that a deviation from the symmetric domain volume drives the fabrication of curved interfaces. However, in this work, the MJP represented by APOSS–BPOSS_4_ surprisingly assembled into two-dimensional flat nanosheets in mixed solvents. They further underwent spiral growth into fascinating three-dimensional terraced pyramidal structures through screw dislocation. These were against the common understanding that size balancing of the MJPs is a must to form the flat nanosheet. The fundamental understanding of the phase separation of highly asymmetric MJPs in solution as well as the manipulation of their crystallization through screw dislocation growth awaits exploration.

## Results and discussion

### Self-assembly of dendron-shaped AB_4_ macromolecules in mixed solvents

**Fig. 1:**
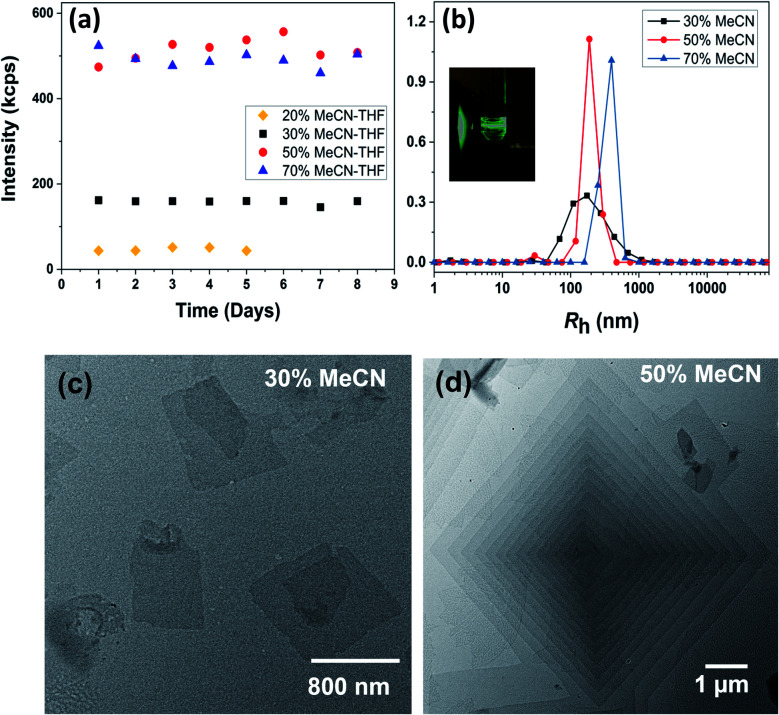
(a) Scattered light intensity and (b) *R*_h_ distributions of 0.2 mg mL^−1^ APOSS–BPOSS_4_ in the THF/MeCN mixed solvent at a 90 degree scattering angle with different MeCN fractions. Inset: Tyndall scattering from the solution observed *via* a green laser. (c and d) TEM images of nanosheets obtained from 0.2 mg mL^−1^ APOSS–BPOSS_4_ in (c) 30 v/v% MeCN/THF and (d) 50 v/v% MeCN/THF, respectively.

The sizes of the BPOSS (*D* ∼ 1.1 nm) and APOSS (*D* ∼ 1.3 nm) cages are comparable,^64^ and APOSS–BPOSS_4_ possesses a high degree of asymmetry with a BPOSS to APOSS volume ratio of ∼3.3 : 1 (*V* = *M*/*ρ*).^49^ The macromolecule APOSS–BPOSS_4_ is soluble in solvents of moderate or low polarity such as THF (solubility parameter *δ* = 18.3 MPa^1/2^), as indicated by the low scattered light intensity at the solvent level (*I* ≈ 10 kcps, *I*_Benzene_ = 18 kcps) recorded by the laser light scattering (LLS) technique. An increase in the scattered light intensity was observed when the selective solvent acetonitrile (*δ* = 24.3 MPa^1/2^), which does not dissolve BPOSS but dissolves APOSS, was introduced into the dilute THF solution of APOSS–BPOSS_4_ at a slow rate of 30 μL h^−1^ (details are presented in ESI†). When the acetonitrile fraction reached 50 v/v%, the scattered intensity immediately increased within the first several hours, suggesting the appearance of large structures. The solution would turn cloudy quickly if the acetonitrile fraction was over 70 v/v%, followed by quick precipitation. However, for the solution containing less acetonitrile, *e.g.*, 20 v/v%, the scattered intensity was relatively low and did not increase over time (Fig. 1a). For the APOSS–BPOSS_4_ solution at the final concentration of 0.2 mg mL^−1^, the average hydrodynamic radii (*R*_h_) measured by the DLS technique at 90 degree scattering angle at 30, 50, and 70 v/v% acetonitrile were 157.1, 192.8, and 356.8 nm, respectively (Fig. 1b), suggesting a growing size with increasing acetonitrile content. The *R*_h_ values show angular dependence, indicating the anisotropic feature of the supramolecular structures in solution (Fig. S4†). The low concentration solutions were stable for the first several days (limited variation in intensity and size) before precipitating in the following weeks.

**Fig. 2:**
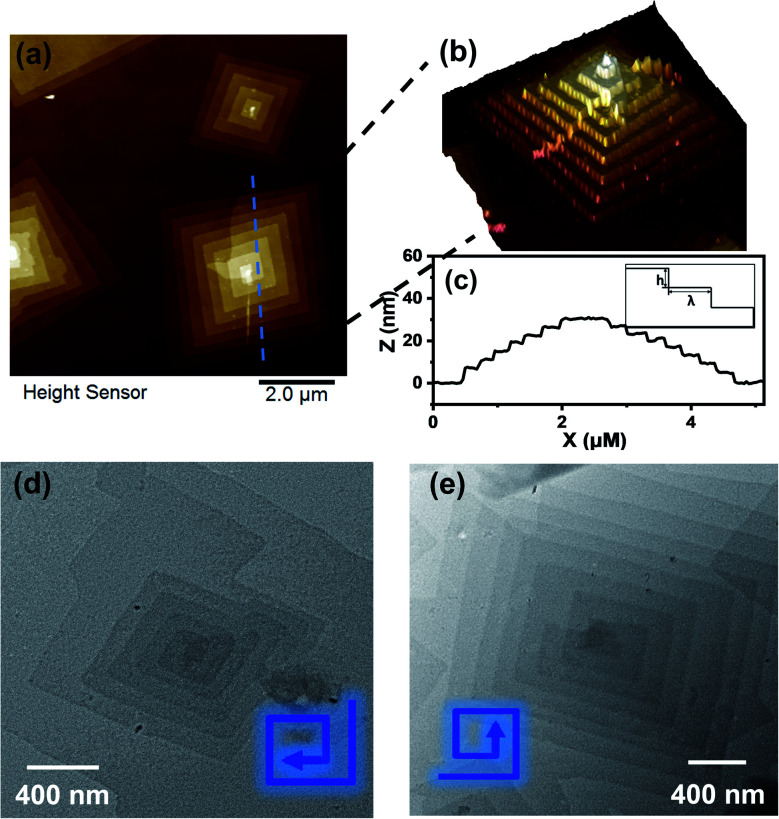
(a and b) AFM images of nanosheets obtained from 0.2 mg mL^−1^ APOSS–BPOSS_4_ in 50 v/v% MeCN/THF. (c) Height analysis of the pyramid-like structure along the dashed lines in image (a), with the inset presenting a side view illustration of the step height (*h*) and terrace width (*λ*) of the multi-layered “pyramids”. (d and e) Left and right-handed screw dislocations, as shown by TEM images.

Transmission electron microscopy (TEM) revealed the assembled morphology as two-dimensional parallelogram nanosheets (Fig. 1c). Very interestingly, some of the nanosheets were observed as terraced in the pyramidal pattern through screw dislocation (Fig. 1d and S6†). Such a pyramidal structure became the majority one when the solutions contained over 50 v/v% of acetonitrile (yield: ∼75% are pyramidal structures and the remaining are 2D nanosheets). The size of the individual or terraced nanosheets ranged from approximately 0.5–7 μm in the lateral distance, measured from the 2D top-view and 3D display. The number of terrace layers observed varied from 2 to 15, and the terrace width (*λ*) was ∼100–300 nm. Histograms presenting the distribution of “pyramid” size and the number of layers are demonstrated in Fig. S5.† According to the atomic force microscopy (AFM) measurements, the thickness of the individual layer and the step height (*h*) of the terrace “pyramids” were both 4.1 ± 0.2 nm on average (Fig. 2a–c). This value is equal to one elemental Burgers vector. Spirals in both left-handed and right-handed directions (Fig. 2d and e) can be observed under an electron microscope with almost equal chance, probably due to the lack of intrinsic chirality in the macromolecule.

**Fig. 3:**
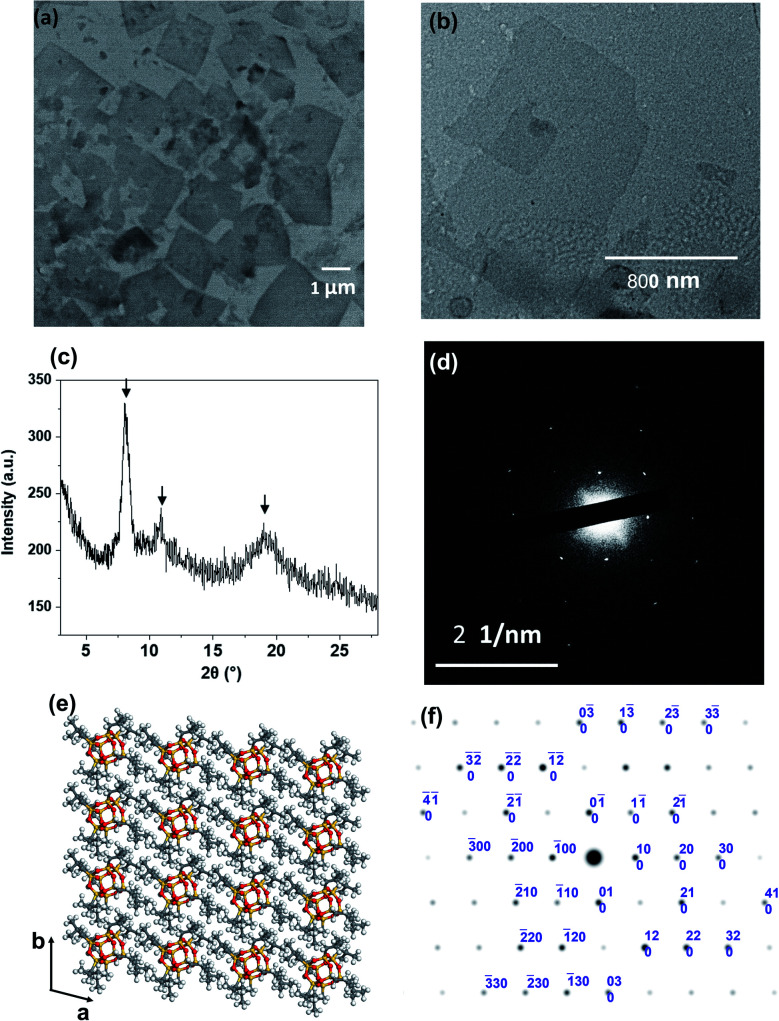
(a) TEM image of the freeze-dried APOSS–BPOSS_4_ nanosheets obtained from 50 v/v% MeCN/THF solution. (b) TEM image of nanosheets dried from 30 v/v% MeCN/THF solution, capturing the onset of dislocation. (c) XRD pattern of the crystallized nanosheets dried from 50 v/v% MeCN/THF solution. The arrows indicate the reflection peaks of BPOSS. (d) SAED pattern of a single-layered APOSS–BPOSS_4_ nanosheet along the [001] zone. (e) Simulation of BPOSS molecular packing in the triclinic crystal lattice on the *ab*-plane. (f) Simulated ED pattern of BPOSS along the [001] zone.

It is crucial to know whether both nanosheet and pyramidal structures co-existed in the solution. Therefore, the freeze-dried sample from 50 v/v% THF/acetonitrile solution was used for TEM imaging. In Fig. 3a, individual 2D nanosheets were still observed, but the pyramidal structure was barely seen. This uncovered that the dendron-shaped macromolecule assembled into 4.1 nm-thick nanosheets in the THF/MECN mixed solution, while some of them further grew into pyramidal structures during fast solvent evaporation at room temperature.

### Molecular packing of AB_4_ macromolecules in 2D nanosheets and the contribution from surface functionality

The collection of the X-ray diffraction (XRD), selected area electron diffraction (SAED), and grazing-incidence small-angle X-ray scattering (GISAXS) patterns revealed the crystallinity of the nanosheet. As shown in Fig. 3c, strong diffraction peaks were observed at 2*θ* = 8.1°, 10.9°, and 19.0°. They match the diffraction peaks of crystalline BPOSS, and the *d* spacing of *ca.* 1.1 nm (at 2*θ* = 8.1°) is in agreement with the dimension of the BPOSS cluster.^65,66^ To verify the formation of BPOSS lamellae, we performed SAED. The SAED pattern of the single-layered APOSS–BPOSS_4_ nanosheet is shown in Fig. 3d, and the dimensions of the unit cell were determined to be *a* = 1.10 nm, *b* = 1.08 nm, and *γ* = 96.1°. As the 3D crystal structure of octaisobutylsilsesquioxane (BPOSS) has already been determined in previous studies,^65^ we notice our determined dimensions to reflect well the key features of the triclinic BPOSS single crystal along the [001] projection. To further confirm our speculation, Accelrys Cerius^2^ software was used to simulate the BPOSS clusters arranged in a 2D lattice with the aforementioned lattice parameters. The crystalline packing arrangements and the corresponding ED pattern are demonstrated in Fig. 3e and f, respectively. By comparing the experimental ED pattern with the computer-simulated spots in terms of both position and intensity, they are found to be in good agreement. This revealed that the formation of APOSS–BPOSS_4_ parallelogram nanosheets was driven by the crystallization of BPOSS when it was oversaturated due to the introduction of the poor solvent acetonitrile, while APOSS was indicated as amorphous. From the GISAXS pattern in Fig. S7(a),† scattering peaks were only observed along the out-of-plane scattering normal to the sample surface. The line intensity scanned along the *q*_z_ direction is plotted as Fig. S7(b).† The primary peak (q*) appeared at *q*_z_ = 0.153 Å^−1^ and the *d*-spacing (*d* = 2π/q*) was calculated to be 4.1 nm, consistent with the thickness of the nanosheet measured from AFM.

It is quite interesting and unexpected to observe that the highly asymmetric APOSS–BPOSS_4_ with the dendron-shaped geometry grows into flat 2D nanosheets in solution for two reasons. First, it was suggested that the size matching of the MJP domains is a major determining factor in the formation of the 2D structures: the MJPs with unbalanced cage sizes such as BPOSS–AC_60_ failed to form regular 2D nanosheets, but facetted 2D nanocrystals can be obtained when one more BPOSS was added to the hybrid to make the overall interfacial area comparable.^64^ Second, previous studies of some similar AB_*n*_ MJPs or traditional block copolymers in the bulk state indicated that the asymmetric topology tends to form curved structures, such as hexagonal cylindrical (HEX), body-centered cubic (BCC), or spherical motifs composed of F–K phases, *etc.*^49,67^ The work herein is the first demonstration of the 2D nanosheets constructed by a dendron-shaped MJP of highly unbalanced size. We assume that the deciding factor for nanosheet crystallization from the dendron-like MJP is its larger overall excluded volume of the crystalline domain (BPOSS) compared to the amorphous part and not the other way around. Besides, the crystallization of AB_*n*_ MJPs in the bulk took place at elevated temperatures, when the crystallization of BPOSS was prevented (at a temperature above *T*_c_), and the closest packing was adopted to adapt the molecular architecture as well as to lower the free energy. Different from the above-discussed points, the mixed solvents used herein showed solvent selection for APOSS and BPOSS, and the crystal growth of BPOSS was also facilitated by the selection of mixed solvents at room temperature. Thus, the increasing acetonitrile volume lowered the effective volume fraction of BPOSS and drove the phase separation. If acetonitrile is not involved, APOSS–BPOSS_4_ would gather into spherical aggregates after evaporation of its THF solvent (Fig. S8†).

In addition to the 2D nanosheets, the 3D nanocrystals growing along the layer normal direction could be observed when the solution underwent slow evaporation in a sealed container (Fig. S9†), indicating that the existence of 2D nanosheets was attributed to its low concentration and faster BPOSS crystal growth in the *X*–*Y* direction compared to the *Z* direction in solution.

**Fig. 4:**
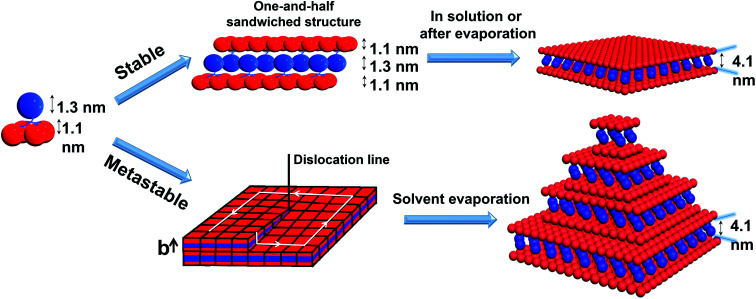
Cartoon illustrations of the proposed mechanisms of APOSS–BPOSS_4_ forming into 2D nanosheets and multi-layered pyramidal structures through screw dislocation.

To pack the dendron-shaped APOSS–BPOSS_4_ into 2D nanosheets, the one-and-half sandwiched structure shown in Fig. 4 is considered as the molecular packing model: APOSS cages form an interdigitated layer, sandwiched by two BPOSS layers. In such a way, the size asymmetry was balanced to maximize the free volume, and the theoretical thickness would be close to the measured thickness of 4.1 nm. The double-layered arrangement is excluded here due to its mismatching thickness: it is impossible to vertically pack four POSS cages (two with 1.1 nm-diameter and two with 1.3 nm-diameter) into 4.1 nm.

**Fig. 5:**
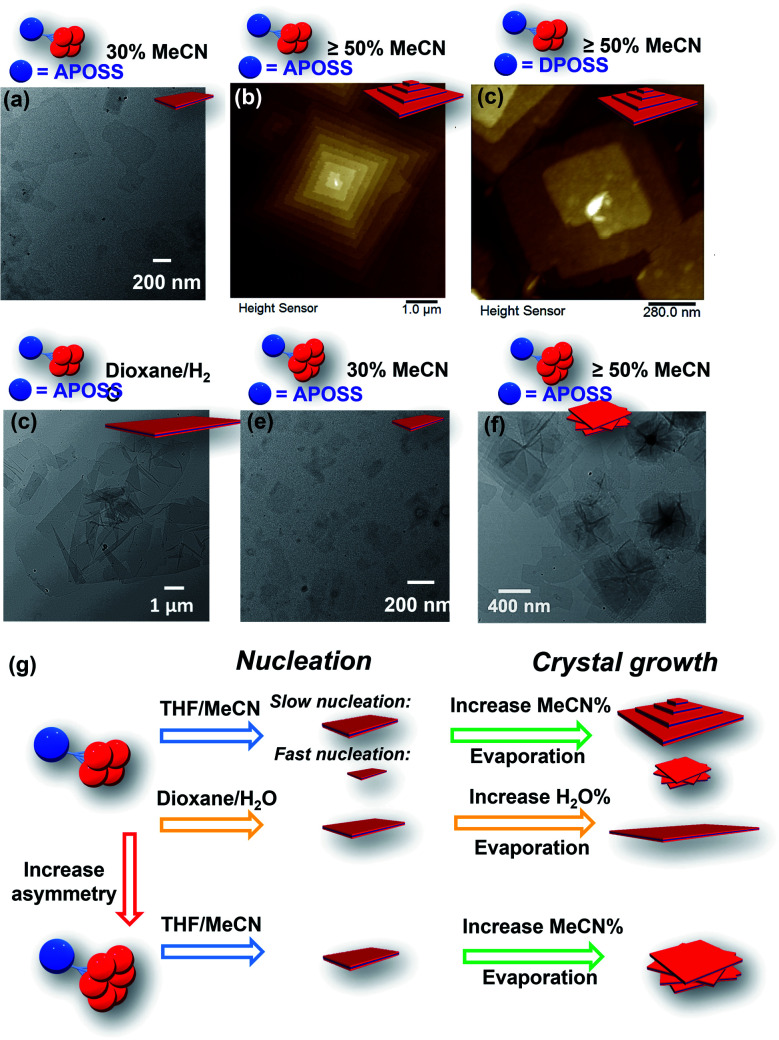
Assembly morphologies and their cartoon illustrations of AB_*n*_ (*n* = 4 or 6) under different conditions: (a) TEM image of APOSS–BPOSS_4_ crystallized into single-layered nanosheets from 30 v/v% MeCN/THF. (b and c) AFM images of (b) APOSS–BPOSS_4_ and (c) DPOSS–BPOSS_4_ crystallized into terraced nanosheets from 50 v/v% MeCN/THF. (d) TEM image of APOSS–BPOSS_4_ crystallized into single-layered nanosheets from 20 v/v% H_2_O/dioxane. (e and f) TEM image of APOSS–BPOSS_6_ crystallized into nanosheets from 30 v/v% MeCN/THF and rotational nanosheets from 50 v/v% MeCN/THF, respectively. (g) Illustration of crystal nucleation and growth affected by supramolecular structures and solvent compositions.

It was also found that the H-bonding between APOSS cages may be important in facilitating the intermolecular association into 2D nanosheets in solution: replacing the APOSS head by the H bond-deficient VPOSS (with vinyl groups) failed to generate supramolecular structures in solution even at high concentrations and high acetonitrile fractions (the scattered light intensity was very low: *I* = 48 kcps from 1.5 mg mL^−1^ solution at 70 v/v% MeCN/THF). However, replacing with the hydroxyl-rich DPOSS exhibited similar phenomena as APOSS–BPOSS_4_ in both solution and dry states (Fig. 5c and S10†). Comparing the FT-IR spectra of the DPOSS–BPOSS_4_ nanosheets with the single macromolecule (Fig. S11†), the absorption peak representing the O–H stretch became broadened and shifted from 3372 cm^−1^ to 3357 cm^−1^, indicating stronger directional H-bonding between DPOSS cages within the sandwiched layer. One concern about the one-and-half layered model may be the repulsion from the carboxylic acids of APOSS cages in the middle layer. The ATR-FTIR spectra indicate that the carboxyl groups were in the protonated state, while the ionized state (COO^−^) was missing (Fig. S12†), so there would not be extra tension from the middle layer to destroy the sheet flatness.

### Generation of pyramidal supramolecular structures due to screw dislocation

According to the molecular packing model proposed above, a large surface area of BPOSS was exposed to the outside. To further decrease the surface energy during the solvent evaporation, screw dislocation easily happened from the screw defects in the 2D nanosheets and grew along the layer normal direction to generate the terraced morphology (Fig. 4). Under low supersaturation conditions, the screw-dislocation-driven mechanism^68^ is regarded as a favorable crystal growth pathway compared to the layer-by-layer (LBL) growth and dendritic growth.^10,15,69,70^ In this way, the crystal can grow without the nucleation of new layers which induces an energy barrier.

The spiral grows from a slip plane where the defects accumulate. Since the crystallization and the generation of defects were not largely promoted at low acetonitrile concentrations, an early-stage screw dislocation was captured from 30 v/v% MeCN/THF solution, as shown in Fig. 3b. A slip plane at the bottom layer is also clearly seen in Fig. 2b, where the screw dislocation started and then the self-penetrating step edges provided by the defects worked as nuclei for new layers to grow on top of the bottom one. Since the bottom layers were formed earlier and had a longer time to grow, they were the largest in lateral size. As the dislocation core spiraled up, the size of the upper layer became smaller and thus the terraced pyramidal structures were formed.

The incidence of screw dislocation defects may be caused by the localized stresses during nucleation.^71,72^ Such stress can be usually brought about by restriction on molecular mobility or a high vapor pressure.^73–75^ For these dendron-shaped highly asymmetric macromolecules, their bulkiness and self-constraint may contribute to the stress and result in some of them not following in-plane-growth when crystallizing. This assumption is indicated by the discovery that such defect induced screw dislocation was not observed for various non-bulky molecular Janus particles such as dumbbell-shaped APOSS–BPOSS;^64^ however, it shows up when it comes to APOSS–BPOSS_4_, and the defect density becomes higher when the molecular asymmetry extends to APOSS–BPOSS_6_ (will be discussed later). In addition to the defects during nucleation, new faces exposed from crevices, collisions, broken (or incomplete) crystals, *etc.* are also regarded as the origin of screw dislocations.^76^ They are likely to happen when nuclei are deposited onto substrates during solvent evaporation.

### Effect of molecular asymmetry, solvent compositions, and assembly kinetics on the supramolecular structures

We were curious to know the impact of an even bulkier molecular architecture on supramolecular structures. Therefore, the size asymmetry of the macromolecule was expanded to APOSS–BPOSS_6_ (*V*_BPOSS_ : *V*_APOSS_ = 5.5 : 1). Upon slow addition of acetonitrile into its THF solution, small-sized single-layered nanosheets were also observed from the solution containing 30 v/v% acetonitrile (Fig. 5e). When increasing the acetonitrile fraction to 50 v/v% or higher, polycrystals were observed with nanosheets showing a rotational mismatch between layers (Fig. 5f and S13†). This suggests more defects in the nuclei, induced by the space crowdedness and the intramolecular strain when accommodating BPOSS cages in one flat plane (Fig. 5g). The SAED pattern revealed no crystallographic correlation among the layers. The average thickness of the individual layer was measured to be 4.2 ± 0.3 nm, close to that of APOSS–BPOSS_4_ nanosheets.

The chance of defects inducing pyramidal structures can be regulated by the choice of solvent components as well as the fraction ratio of mixed solvents (Fig. 5g). We have shown that the 2D flat nanosheets were dominant at low acetonitrile fractions (*i.e.*, 30%), while the pyramidal structures were induced by increasing acetonitrile (Fig. 5a, b, e, and f). In addition to this regulator, non-volatile solvents played the same role. When the solvent components THF/MeCN were replaced by slowly titrating a small amount of H_2_O (less than 20%) into the 1,4-dioxane solution of APOSS–BPOSS_4_, nanosheets in “pyramid-like” morphology were not likely to be observed. Instead, nanosheets (thickness = 4.1 ± 0.2 nm) of super large surface area with the lateral size up to 8 microns were obtained from the slow mixing solution (Fig. 5d and S15†). This might be due to the slow evaporation rate of the 1,4-dioxane/H_2_O system that provides the crystalline motif with enough time to undergo stable growth into flat nanosheets.

Adjusting the solvent mixing rate is expected to affect the nucleation kinetics as well as the crystal growth, thereby controlling the size of crystals and the appearance of spiral growth. It was stated previously that under slow nucleation (by introducing acetonitrile at a slow rate), a higher fraction of acetonitrile increased the size of flat and terraced nanosheets gained from the APOSS–BPOSS_4_ solution. However, this trend was reversed when acetonitrile was quickly titrated in one shot into the solution: the supramolecular structures were smaller at a higher acetonitrile fraction, dropping to below 100 nm from DLS measurements. The measured lateral sizes of nanosheets also dropped significantly, to below micron levels at 70 v/v% acetonitrile (Fig. S14†). Moreover, polycrystals with rotational nanosheets along the *Z* direction were also observed, indicating that a higher density of defects during the nucleation results from a faster nucleation rate (Fig. 5g). Herein, solvent mixing rates and solvent compositions can act as regulators of screw dislocation, in addition to other regulating factors, such as pressure or temperature.

## Conclusions

In summary, we explored the hierarchical crystallization of dendron-shaped molecular Janus particles from solution. The APOSS–BPOSS_4_ macromolecule with a high degree of topological anisotropy grew into 2D flat nanosheets from mixed solvents, in contrast to curved structures in the bulk. One-and-half sandwiched molecular packing was adopted to fit the unbalanced macromolecular shape. It was found that the presence and dominance of the crystalline part in the unbalanced topology, solvent selection, and relatively low temperature (RT) were the keys to generate the flat structure instead of the curved one. Because of the bulkiness of the molecular structure, defects were easily generated in nanosheets, which facilitated their spiral growth into well-ordered pyramid-like structures through screw dislocation during solvent evaporation. The generation of spiral structures can be promoted by increasing the acetonitrile fraction to its THF solution or inhibited by using non-volatile mixed solvents. This work highlighted the role of molecular topology and solvent selection in the crystallization of highly asymmetric macromolecules, which would further inspire the supramolecular design and manipulation.

## Author contributions

X. S. and X. F. designed the experiments and prepared the manuscript. X. F. and R. Z. conducted the synthesis. X. S. prepared the samples and conducted characterization experiments. X. F. and X. Y. carried out the diffraction analysis and simulation. T. L. conducted the GISAXS experiments. J. L., H. L., J. C., F. L., and E. R. participated in the characterization experiments, data analysis, and discussion. The authors declare no competing financial interest.

## Conflicts of interest

The authors declare no competing financial interest.

## Supplementary Material

SC-012-D1SC02617H-s001
