# Supplementary material for: Screw dislocation-induced pyramidal crystallization of dendron-like macromolecules featuring asymmetric geometry
Source: Chem Sci. 2021 Aug 6;12(36):12130–7. doi: 10.1039/d1sc02617h (PMC8459181; doi:10.1039/d1sc02617h)
Supplement: SC-012-D1SC02617H-s001 [file SC-012-D1SC02617H-s001.pdf]

Electronic Supplementary Material (ESI) for Chemical Science.
This journal is © The Royal Society of Chemistry 2021

Supporting information

Screw Dislocation-Induced Pyramidal Crystallization of Dendron-Like Macromolecules Featuring Asymmetric Geometry

Xinyu Sun[†], Xueyan Feng[†], Xiao-Yun Yan[‡], Jiancheng Luo[†], Ruimeng Zhang[†], Tao Li[§], Hui Li[†], Jiahui Chen[†], Fangbei Liu[†], Ehsan Raee[†], Stephen Z.D. Cheng[*,†,‡], Tianbo Liu[*,†]

[†]School of Polymer Science and Polymer Engineering, The University of Akron, Akron, Ohio 44325, United States

[‡]South China Advanced Institute for Soft Matter Science and Technology, School of Molecular Science and Engineering, South China University of Technology, Guangzhou 510640, China

[§]X-Ray Science Division, Advanced Photon Source, Argonne National Laboratory, Argonne, IL 60439, United States

Table of Contents

1. **Figure S1.** Cartoons and chemical structures of AB_n dendron-like molecules based on POSS nanoparticles.
2. **Synthetic procedures**
3. **Sample preparation and characterizations**
4. **Figure S2.** ^{1}H NMR spectra of: (a) APOSS-BPOSS$_4$, (b) APOSS-BPOSS$_6$
5. **Figure S3.** ^{13}C NMR spectra of: (a) APOSS-BPOSS$_4$, (b) APOSS-BPOSS$_6$
6. **Figure S4.** The R_h of the 0.2 mg/mL APOSS-BPOSS$_4$ assembly in 60% v/v MeCN/THF solution measured at different scattering angles.
7. **Figure S5.** Histograms presenting the distribution of (up) size and (down) layer number of the pyramidal structures.
8. **Figure S6.** TEM images of pyramid-like nanosheets obtained from 0.2 mg/mL APOSS-BPOSS$_4$ in (a) 50 v/v% MeCN/THF and (b) 70 v/v% MeCN/THF, respectively.
9. **Figure S7.** (a) 2D GISAXS pattern and (b) vertical intensity cut profile.
10. **Figure S8.** TEM image of APOSS-BPOSS$_4$ dried from THF solution.
11. **Figure S9.** 3D nanocrystals observed from solution slowly evaporated in a sealed container.
12. **Figure S10.** (a) TEM image, (b) SAED pattern, and (c) AFM image of DPOSS-BPOSS$_4$ in 50 v/v% MeCN/THF.
13. **Figure S11.** Up: ATR-FTIR spectra of DPOSS-BPOSS$_4$ in single molecule state (black curve) and crystallized nanosheet state (red curve). Down: An estimation of distances between DPOSS cages in the middle layer.
14. **Figure S12.** ATR-FTIR spectra of APOSS-BPOSS$_4$ in 50 v/v% MeCN/THF solution and dry state.
15. **Figure S13.** (a) Scattered light intensity and (b) R_h distributions of APOSS-BPOSS$_6$ in THF/MeCN mixed solvents. The R_h distributions in (b) are R_h = 164.0 nm and 135.9 nm for 50 v/v% MeCN/THF and 70 v/v% MeCN/THF, respectively. (c-d) TEM iamges, SAED pattern of (d), and (f) AFM images of APOSS-BPOSS$_6$ obtained from 50 v/v% MeCN/THF. (g) The height profile along the dash lines in (f).
16. **Figure S14.** (a) Scattered light intensity, (b) R_h distributions of APOSS-BPOSS$_4$ in THF/MeCN mixed solvents, and (c) corresponding TEM images through fast nucleation. The R_h distributions are R_h = 92.8 nm and 45.9 nm for 50 v/v% MeCN/THF and 70 v/v% MeCN/THF, respectively. The conditions are (c) 50 v/v% MeCN/THF and (d-e)70 v/v% MeCN/THF.
17. **Figure S15.** (a-b) TEM images and (c) AFM image of APOSS-BPOSS$_4$ obtained from (a,c) 20 v/v% and (b) 30 v/v% H_2O/dioxane mixed solvents. (d) The height profile along the dash line in (c). The thickness of the single layer was 4.1 ± 0.2 nm in average.

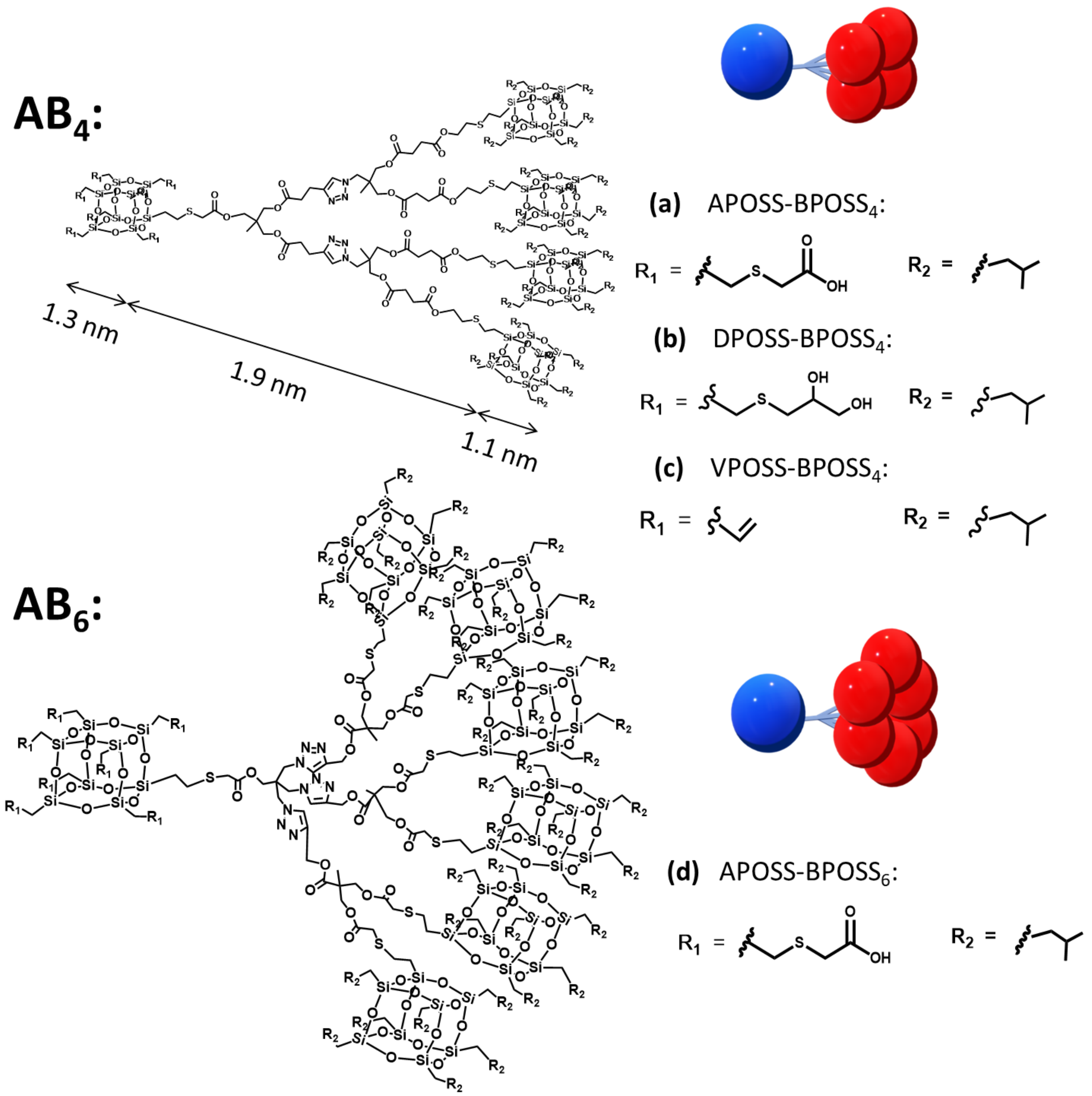

Figure S1. Cartoons and chemical structures of AB_n dendron-like molecules based on POSS nanoparticles: (a) APOSS-$BPOSS_4$; (b) DPOSS-$BPOSS_4$; (c) VPOSS-$BPOSS_4$; (d) APOSS-$BPOSS_6$.

Materials

OctaVinylPOSS, allylIsobutyl POSS, vinylisobutyl POSS were purchased from Hybrid plastics, Cuprous bromide (CuBr, Aldrich, 98 %) was freshly purified by stirring in acetic acid overnight, washed with acetone, and dried in vacuum. VPOSS-3N_3,[1] Yne-2OH,[2] VPOSS-$BPOSS_4$,[3] and DPOSS-$BPOSS_4$[3] were synthesized as reported. All other chemicals were purchased from Sigma-Aldrich and solvents were filtered before usage.

Synthetic procedures

The AB_n type molecules were constructed via esterification reactions, copper catalyzed azide–alkyne cycloaddition (CuAAC) “click” chemistry, and thiol-ene reaction following the similar routes reported previously.[3,4]

As an example, VPOSS-$BPOSS_4$ was synthesized via “click” chemistry using 1 equiv of VPOSS-2Yne and 2 equiv of 2BPOSS-N_3 as shown in Scheme 1. Afterwards, VPOSS in the VPOSS-$BPOSS_4$ sample can be further modified to be hydrophilic through thiol-ene reaction. The detailed synthesis and characterization information were provided in *ACS Central Science* 2017, 3, 860-867.

Scheme 1. Synthetic routes of VPOSS-BPOSS$_4$ and its precursors:

(ia) 1,1,1-Tris(hydroxymethyl)ethane, DIPC, DMAP, THF, 60%; (ib) 4-Pentynoic acid, DIPC, DMAP, THF, 69%;

(iia) 2-1 Methyl-2-propanethiol, Irgacure 2959, 2 THF, 10min, 91%; (iib) succinic anhydride, TEA, DMAP, THF, 91%; (iic) 2-(azidomethyl)-2-methylpropane-1,3-diol, DIPC, DMAP, THF, 79%;

(iiia) CuBr, PMDETA, THF, 94%;

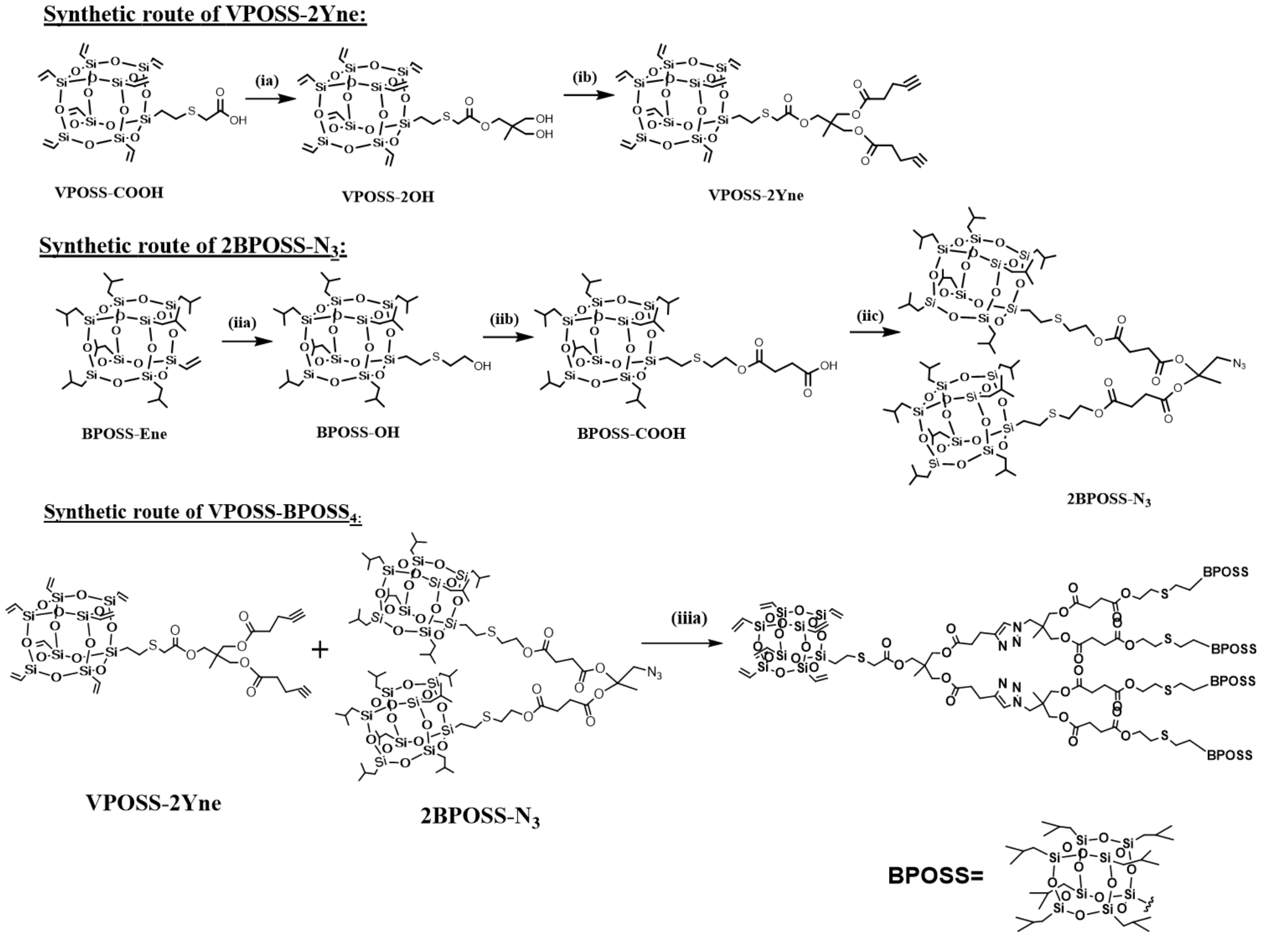

Similarly, VPOSS-BPOSS$_6$ was synthesized via “clicking” 1 equiv of VPOSS-3N$_3$ and 3 equiv of 2BPOSS-Yne as shown in Scheme 2. VPOSS in the VPOSS-BPOSS$_6$ sample can be further modified to be hydrophilic via thiol-ene reaction. The detailed synthesis and characterization information were provided as following.

Scheme 2. Synthetic routes of VPOSS-BPOSS$_6$ and its precursors:
(i) photoinitiator Irgacure 2959, 365nm UV light for 15 min, THF, 95%; (ii) DIPC, DMAP, THF, 80%; (iii) CuBr, PMDETA, THF, 92%.

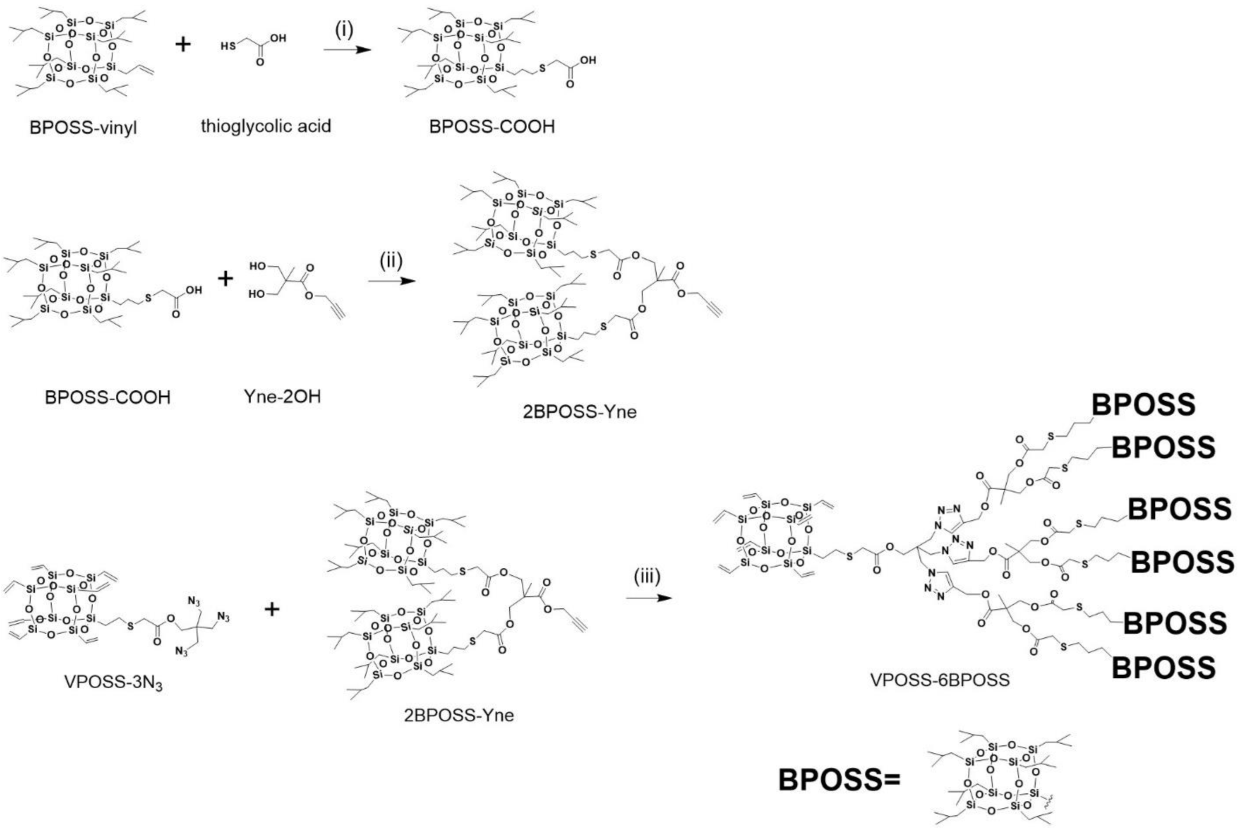

Synthesis of compound BPOSS-COOH

In an uncapped 20ml vial equipped with a stirring bar, allylIsobutyl POSS (BPOSS-vinyl, 1.0 g, 1.2 mmol), thioglycolic acid (290 mg, 3.2 mmol) and photoinitiator Irgacure 2959 (3 mg) were added, followed by the addition of 10ml THF to completely dissolved the solids. The mixture was applied to 365nm UV light for 15 min. Then THF was evaporated under vacuum. The residue was purified by chromatography on silica gel using CH_2Cl_2:EA=20:1 as the eluent to acquire the product (Yield: 95%). ^{1}H NMR ($CDCl_3$, 300 MHz, ppm): δ = 3.26 (s, 2H, -S-C$\underline{H}_2$-CO), 2.68 (t, 2H, -CH_2-C$\underline{H}_2$-S-CH_2-CO-), 1.96-1.66 (m, 9H, -Si-CH_2-C$\underline{H}_2$-CH_2-S-, -Si-CH_2-C$\underline{H}$(CH_3)$_2$), 0.97 (d, 42H, -Si-CH_2-CH(C$\underline{H}_3$)$_2$), 0.73 (t, 2H, -Si-C$\underline{H}_2$-CH_2-CH_2-S-), 0.62 (q, 14H, -Si-C$\underline{H}_2$-CH(CH_3)$_2$).

Synthesis of compound 2BPOSS-Yne

In a 100 mL round-bottom flask with a magnetic stirring bar, Yne-2OH (30.2 mg, 0.18 mmol), BPOSS-COOH (500 mg, 0.53mmol) and DMAP (4.2 mg, 0.03 mmol) were fully dissolved in 20 mL freshly dried THF. The mixture was capped by a rubber septum, cooled to 0 ^{o}C and stirred at that temperature for 10 min, and then DIPC (67 mg, 0.53 mmol) was added dropwise via syringe. The mixture was warm up to room temperature and stirred for another 24 h. After that, the white precipitate was filtered off, and the filtrate was evaporated under vacuum. The residue was purified by flash column chromatography on the silica gel with CH_2Cl_2/EA (v/v = 80/1) as eluent to afford final product (Yield: 80%). ^{1}H NMR ($CDCl_3$, 300 MHz, ppm): δ = 4.73 (d, 2H, Alkyne-$C\underline{H}_2$-O-), 4.31 (s, 4H, -$C\underline{H}_2$-O-CO-CH2-S-), 3.23 (s, 4H, -CO-$C\underline{H}_2$-S-), 2.71 (t, 4H, -CH_2-$C\underline{H}_2$-S-CH_2-CO-), 2.46 (t, 1H, $C\underline{H}$≡C-), 1.98-1.70 (m, 18H, -Si-CH_2-$C\underline{H}_2$-CH_2-S-, -Si-CH_2-$C\underline{H}(CH_3)_2$), 0.96 (m, 87H, -$C\underline{H}_3$), 0.72-0.57 (m, 32H, -Si-$C\underline{H}_2$-CH_2-CH_2-S-, -Si-$C\underline{H}_2$-$CH(CH_3)_2$).

Synthesis of compound VPOSS-BPOSS$_6$

VPOSS-3N_3 (30 mg, 0.03 mmol), 2BPOSS-Yne (266 mg, 0.13 mmol), CuBr (5mg, 0.04 mmol) were totally dissolved in 20 ml dry THF in a Schlenk flask and degassed for three times. Then PMDETA (15mg, 0.09 mmol) was added. The system was degassed again and stirred for 12 h. After the reaction was completed, the solution was added into silica gel column. THF was applied to elute off the crude product. After solvent removal, silica gel column chromatography using CH_2Cl_2 as the eluent was applied to purify the residue and acquire the product (Yield: 92%). ^{1}H NMR ($CDCl_3$, 300 MHz, ppm): δ = 8.16 (s, 3H, Triazole), 6.17-5.80 (m, 21H, -$C\underline{H}$=$C\underline{H}_2$), 5.27 (s, 6H, -CO-O-$C\underline{H}_2$-Triazole), 4.53 (s, 6H, -C-$C\underline{H}_2$-Triazole), 4.30 (q, 12H, -$C\underline{H}_2$-O-CO-CH_2-S-CH_2-CH_2-CH_2-BPOSS), 3.70 (s, 2H, -$C\underline{H}_2$-O-CO-CH_2-S-CH_2-CH_2-VPOSS), 3.35 (s, 2H, -CH_2-O-CO-$C\underline{H}_2$-S-CH_2-CH_2-VPOSS), 3.18 (s, 12H, BPOSS-CH_2-CH_2-CH_2-S-$C\underline{H}_2$-CO-), 2.83 (t, 2H, -S-$C\underline{H}_2$-CH_2-VPOSS), 2.62 (t, 12H, BPOSS-CH_2-CH_2-$C\underline{H}_2$-S-CH_2-CO-), 1.98-1.60 (m, 54H, -Si-CH_2-$C\underline{H}_2$-CH_2-S-, -Si-CH_2-$C\underline{H}(CH_3)_2$), 1.02-0.93 (m, 263H, -$C\underline{H}_3$, -Si-$C\underline{H}_2$-CH_2-S-), 0.77-0.57 (m, 96H, -Si-$C\underline{H}_2$-CH_2-CH_2-S-, -Si-$C\underline{H}_2$-$CH(CH_3)_2$). ^{13}C NMR ($CDCl_3$, 500 MHz, ppm): δ = 172.12, 169.80, 168.50, 142.28, 137.08, 128.56, 127.06, 65.65, 58.07, 49.53, 46.62, 45.01, 35.48, 33.05, 25.69, 23.86, 22.48, 22.35, 17.58, 11.46.

Synthesis of compound APOSS-BPOSS$_6$

VPOSS-BPOSS$_6$ (100 mg, 0.014 mmol), Thioglycolic acid (18mg, 0.20 mmol), and the photoinitiator Irgacure 2959 (3 mg) were dissolved in 3 mL THF in an open vial with a stirring bar. The solution was conducted under irradiation by 365 nm UV light for 10 min. The product was purified by precipitation into methanol/water 1:3 twice. ^{1}H NMR ($CDCl_3$, 300 MHz, ppm): δ = 8.20 (s, 3H, Triazole), 5.29 (s, 6H, -CO-O-$C\underline{H}_2$-Triazole), 4.56 (s, 6H, -C-$C\underline{H}_2$-Triazole), 4.30 (q, 12H, -$C\underline{H}_2$-O-CO-CH_2-S-CH_2-CH_2-CH_2-BPOSS), 3.77 (s, 2H, -$C\underline{H}_2$-O-CO-CH_2-S-CH_2-CH_2-APOSS), 3.46-3.07 (m, 28H,

$-CH_2-O-CO-C\underline{H}_2-S-CH_2-CH_2-$APOSS, BPOSS$-CH_2-CH_2-CH_2-S-C\underline{H}_2-CO-$, $-S-C\underline{H}_2-COOH$), 2.91-2.49 (m, 28H, $-S-C\underline{H}_2-CH_2-$APOSS, BPOSS$-CH_2-CH_2-C\underline{H}_2-S-CH_2-CO-$), 1.98-1.60 (m, 54H, $-Si-CH_2-C\underline{H}_2-CH_2-S-$, $-Si-CH_2-C\underline{H}(CH_3)_2$), 1.19-0.83 (m, 277H, $-C\underline{H}_3$, $-Si-C\underline{H}_2-CH_2-S-$), 0.77-0.52 (m, 96H, $-Si-C\underline{H}_2-CH_2-CH_2-S-$, $-Si-C\underline{H}_2-CH(CH_3)_2$). ^{13}C NMR ($CDCl_3$, 500 MHz, ppm): δ = 172.22, 169.95, 142.16, 127.21, 65.63, 57.89, 46.53, 35.43, 32.95, 25.66, 23.83, 22.41, 22.27, 17.51, 12.08, 11.39.

Synthesis of compound APOSS-BPOSS$_4$

VPOSS-BPOSS$_4$ (100 mg, 0.019 mmol), Thioglycolic acid (24mg, 0.26 mmol), and the photoinitiator Irgacure 2959 (3 mg) were dissolved in 3 mL THF in an open vial with a stirring bar. The solution was conducted under irradiation by 365 nm UV light for 10 min. The product was purified by precipitation into methanol/water 1:3 twice. ^1H NMR ($CDCl_3$, 300 MHz, ppm): δ =7.49 (s, 2H, Triazole), 4.62-3.89 (m, 24H, $-C\underline{H}_2-O-CO-CH_2-CH_2-$, Triazole(N)$-C\underline{H}_2-$), 3.74 (s, 2H, $-C\underline{H}_2-O-CO-CH_2-S-$), 3.51 (s, 2H, $-S-C\underline{H}_2-CO-O-CH_2-$), 3.28 (s, 14H, $-S-C\underline{H}_2-COOH$), 3.05 (t, 4H, Triazole(C)$-C\underline{H}_2-$), 2.92-2.58 (m, 52H, $-O-CO-C\underline{H}_2-CH_2-$, $-S-C\underline{H}_2-CH_2-$), 1.92-1.79 (m, 28H, $-Si-CH_2-C\underline{H}(CH_3)_2$), 1.17-0.89 (m, 201H, $-C\underline{H}_3$, $-Si-C\underline{H}_2-CH_2-S-$), 0.67-0.56 (m, 56H, $-Si-C\underline{H}_2-CH(CH_3)_2$). ^{13}C NMR ($CDCl_3$, 500 MHz, ppm): δ =172.02, 145.98, 66.02, 63.59, 39.52, 33.30, 30.16, 28.86, 26.58, 25.69, 23.86, 22.44, 22.35, 19.30, 17.85, 13.60, 12.16.

Sample preparation and characterizations:

Sample preparation:

As a typical sample preparation, AB_n powder was dissolved in THF or 1,4-dioxane at the concentration of 5 mg/mL as stock solution. It was filtered and diluted to desired concentrations before usage. A selective filtered solvent (acetonitrile or DI H_2O) was titrated into the solution at the rate of 30 μL/h to trigger the crystallization/assembly. The initial volume of solution varied from 180 to 420 μL, so as to keep the solutions at the same final concentration and volume (600 uL). The solute concentrations mentioned in the text were the final concentration after solvent mixing.

Characterizations:

Solution state Nuclear Magnetic Resonance (NMR) Spectroscopy: ^1H and ^{13}C NMR spectra were obtained in $CDCl_3$ (Sigma-Aldrich, 99.8% D) solvents utilizing Varian Mercury 300 MHz NMR or 500 MHz NMR spectrometer. ^1H NMR spectra were referenced to the residual proton impurities in CDCl3 at δ 7.27 ppm and ^{13}C NMR spectra were referenced to ^{13}CDCl3 at δ 77.00 ppm.

Dynamic Light Scattering (DLS): Experiments were performed on a commercial Brookhaven laser scattering spectrometer that was equipped with a 532 nm laser. An intensity-intensity BI-9000AT correlator was used to get the correlation function based on the particle movement. The diffusion coefficient D could be obtained by $\Gamma = Dq^2$, which Γ was calculated by CONTIN

method. The hydrodynamic radius (R_h) can be calculated according to the Stokes-Einstein equation: $R_h=k_bT/6\pi\eta D$ in which k_b is the Boltzmann constant and η is the viscosity of the solution.

Transmission Electron Microscopy (TEM): The TEM images were taken by a JEOL JEM-1230 electron microscope operated at 120 kV. The samples were prepared by dropping 8 μL of solution onto a carbon-coated copper grid, and the sample was allowed to dry at room temperature overnight. Freeze-dried sample was prepared by quenching the sample in liquid nitrogen for 30 mins and then pump in vacuum under the liquid nitrogen environment for 2 days.[5]

Atomic Force Microscopy (AFM): The AFM experiments were conducted on a Dimension Icon Atomic Force Microscopy (Bruker). Samples were prepared by drop-casting 10 μL onto the acetone rinsed Si wafer substrates. After solvent evaporation under ambient conditions overnight, images were scanned in the tapping mode and analyzed by NanoScope Analysis software.

Grazing-Incidence Small-angle X-ray Scattering (GISAXS). GISAXS experiments were performed at the 12-ID-B beamline of the Advanced Photon Source (APS) at Argonne National Laboratory. The operating conditions were chosen as a wavelength of 1.33 Å and a sample-to-detector distance of 3.63 m.

Attenuated Total Reflectance Fourier Transform Infrared Spectroscopy (ATR-FTIR). A THERMO NICOLET Is-50R FTIR SPECTROMETER equipped with an ATR setup was used to investigate the IR spectrum. 50 μL of the samples were dropped onto the ATR window and tested immediately for 10 times to obtain an average IR spectrum.

X-ray Diffraction (XRD). The crystal structure of the assembled nanosheets was characterized by a Rigaku Ultima IV X-ray diffractometer with Cu Kα radiation (λ= 0.1542 nm). The scanning 2θ angle ranged between 3° and 28° with a step interval of 0.01°.

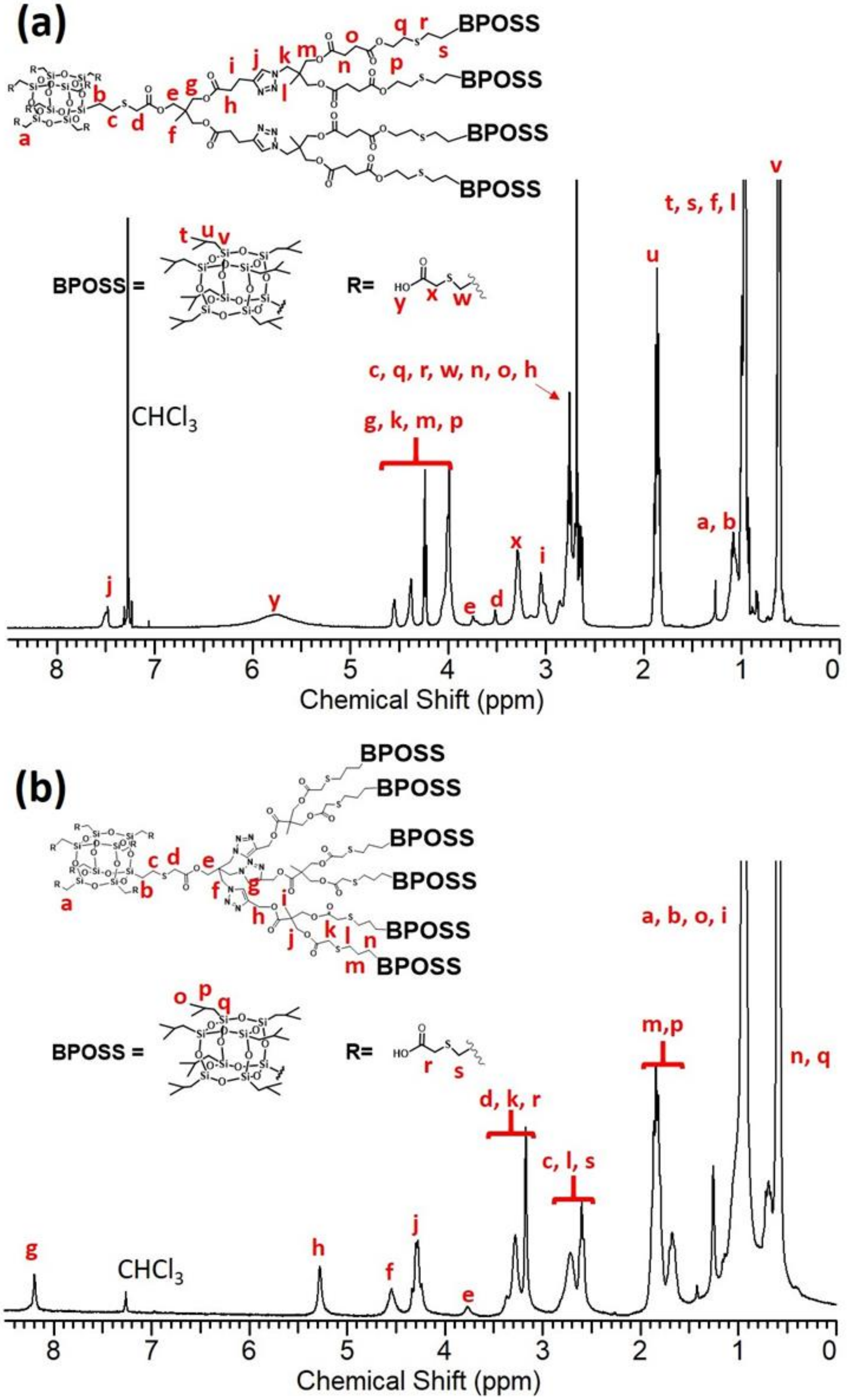

Figure S2. ^{1}H NMR spectra of: (a) APOSS-BPOSS$_4$, (b)APOSS-BPOSS$_6$.

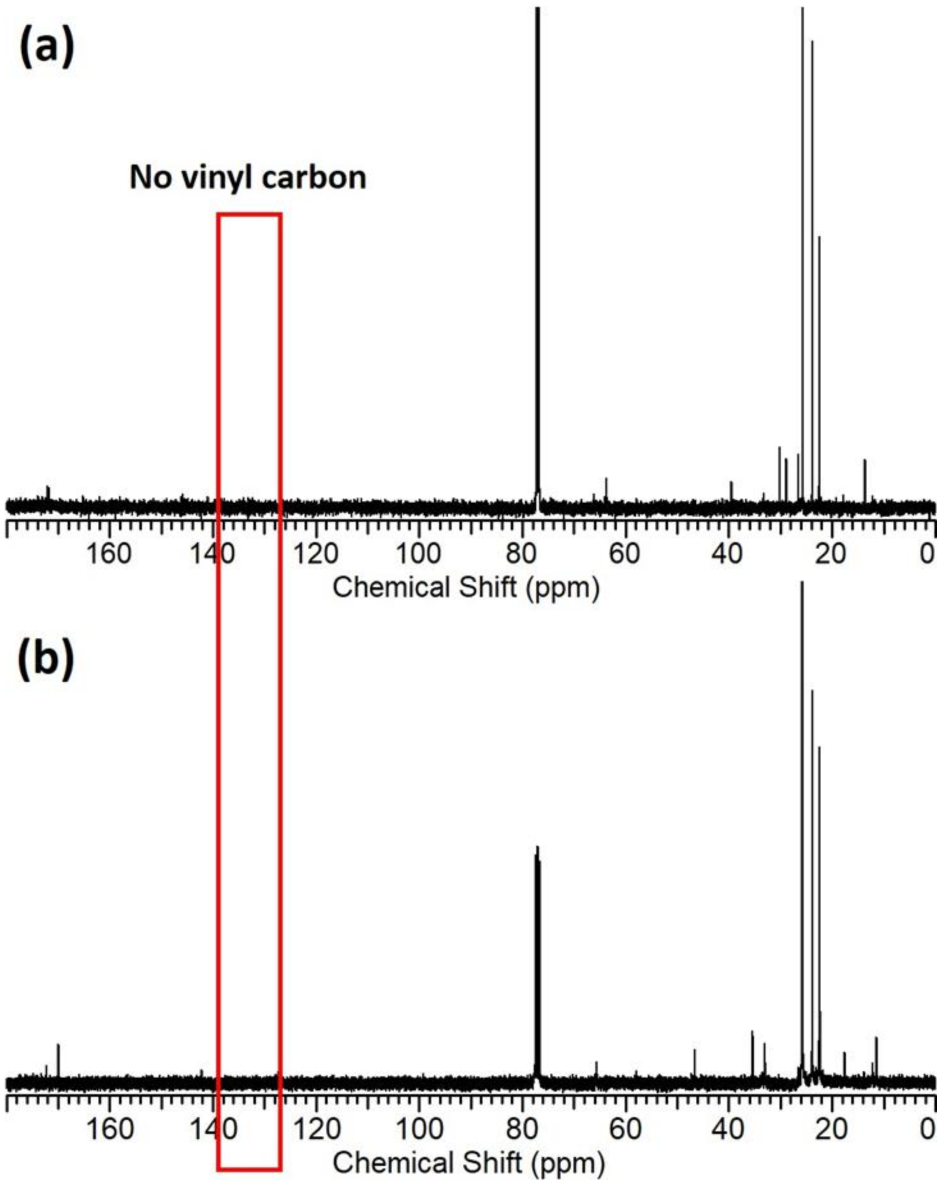

Figure S3. ^{13}C NMR spectra of: (a) APOSS-BPOSS$_4$, (b) APOSS-BPOSS$_6$.

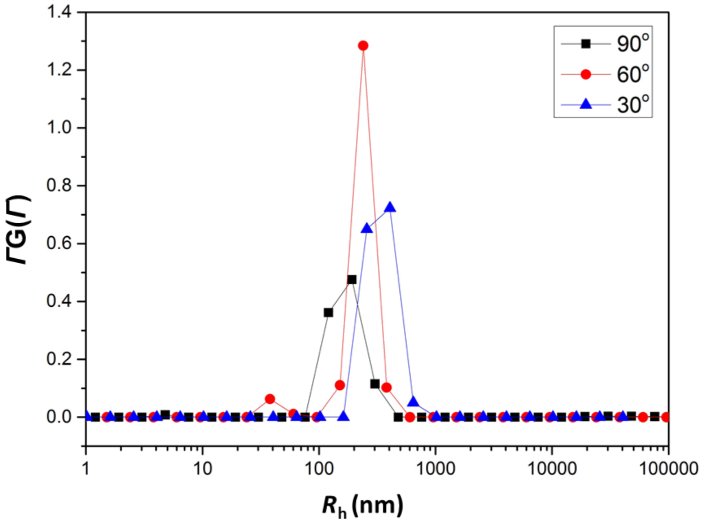

Figure S4. The R_h of the 0.2 mg/mL APOSS-BPOSS$_4$ assembly in 60% v/v MeCN/THF solution measured at different scattering angles, suggesting the angular dependence of the supramolecular structures.

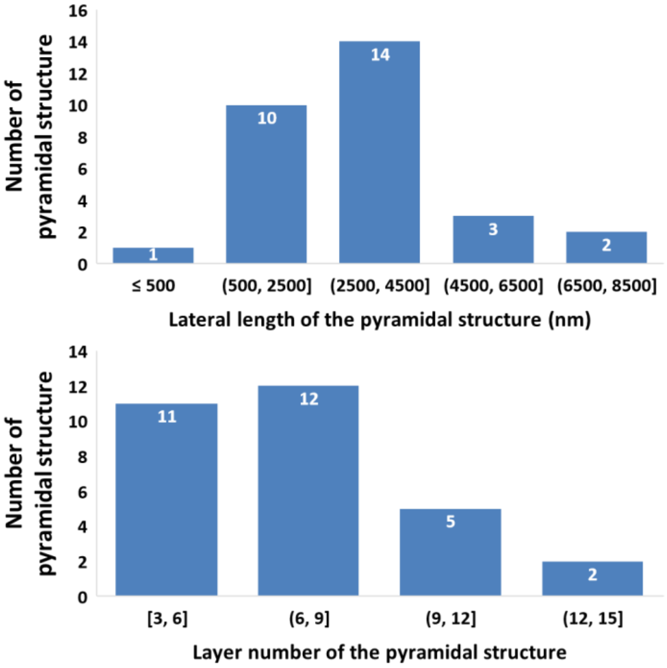

Figure S5. Histograms presenting the distribution of (up) size and (down) layer number of the pyramidal structures. Data were collected by the measurement of 30 "pyramids" from 10 TEM images. Experimental condition: 0.2 mg/mL APOSS-BPOSS$_4$ assembly in 50% v/v MeCN/THF solution.

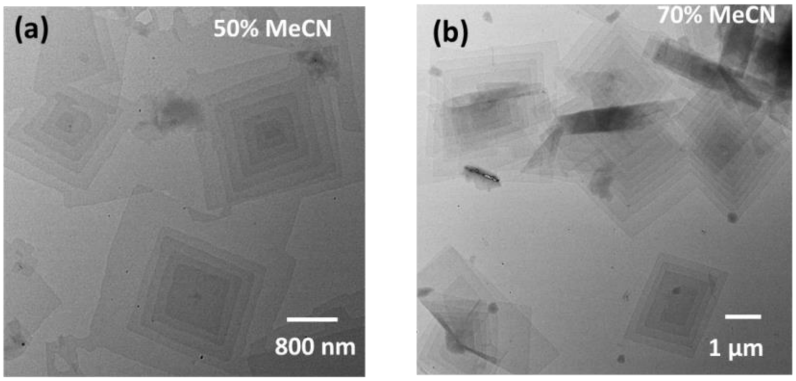

Figure S6. TEM images of pyramid-like nanosheets obtained from 0.2 mg/mL APOSS-BPOSS$_4$ in (a) 50 v/v% MeCN/THF and (b) 70 v/v% MeCN/THF, respectively.

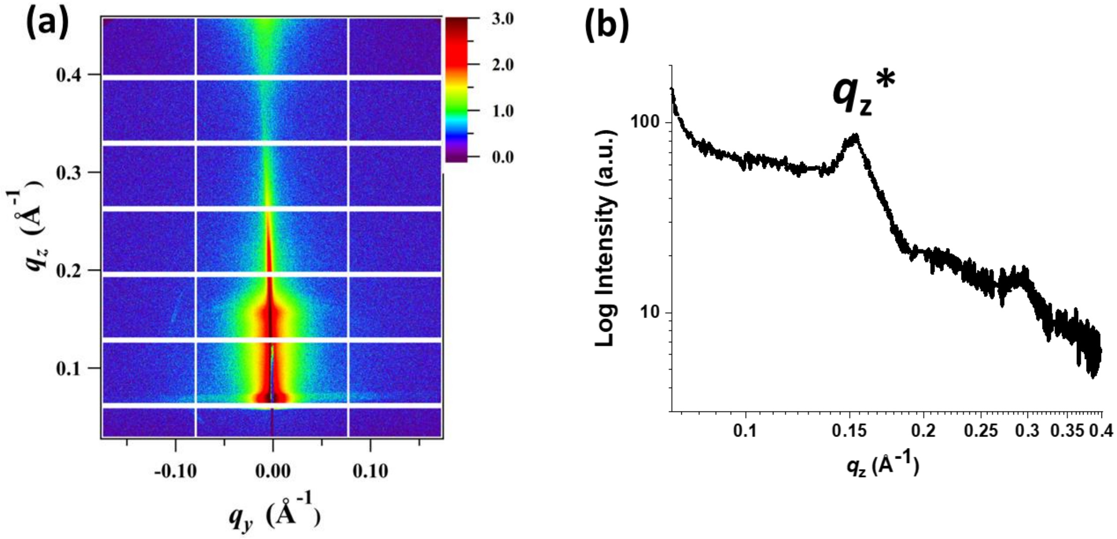

Figure S7. (a) 2D GISAXS pattern and (b) vertical intensity cut profile. In the grazing incidence scattering geometry, α_f and $2\theta_f$ are the exit angles of X-ray beam along the out-of-plane scattering normal to the sample surface and along the in-plane scattering parallel to the sample surface, respectively. The scattering vector q_z is defined by $q_z = (4\pi/\lambda) \sin \alpha_f$, where λ is the wavelength. Two peaks were observed from the GISAXS pattern, with the primary peak (q*) at q_z=0.15 Å^{-1} and a higher order peak at q_z=0.30 Å^{-1}. The ratio of q values (q/q*) are 1, 2, which is consistent with that of the Lamella structure.

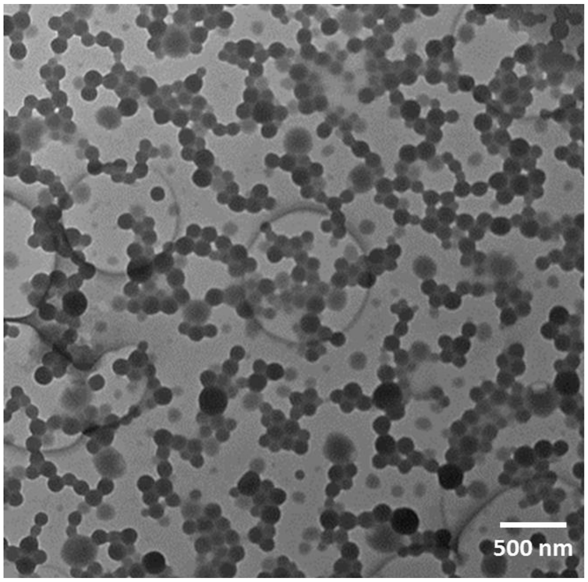

Figure S8. TEM image of APOSS-BPOSS$_4$ dried from THF solution.

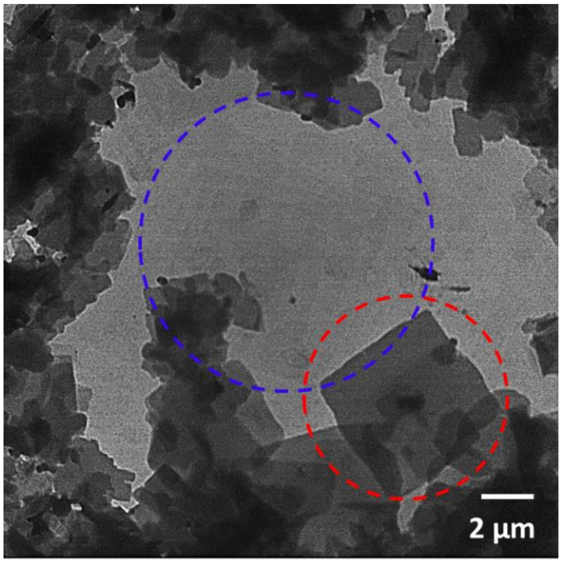

Figure S9.3D nanocrystals observed from solution slowly evaporated in a sealed container. 3D Nanocrystals (in red circle) grown along the layer normal direction have a much greater thickness and therefore showing a greater contrast compared to pyramidal structures (in blue circle) in TEM images. The solution condition was 1.0 mg/mL APOSS-BPOSS$_4$ in 50 v/v% MeCN/THF mixed solvent.

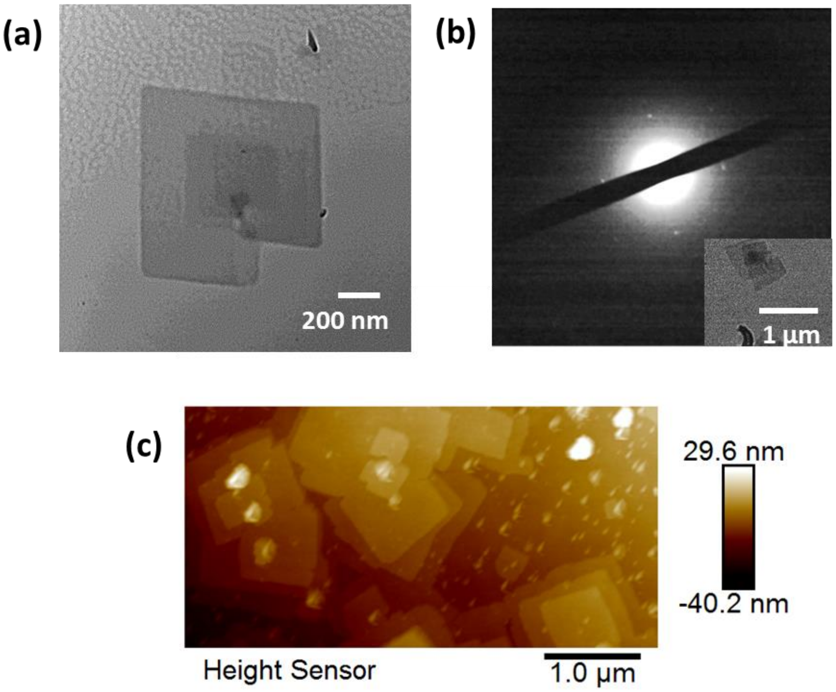

Figure S10. (a) TEM image, (b) SAED pattern, and (c) AFM image of DPOSS-BPOSS$_4$ in 50 v/v% MeCN/THF. For this AFM imaging, the surface of the Si wafer substrate was treated with piranha solution before sampling. This treatment intended to make super hydrophilic surface with a contact angle of ~5°,[6] while that of the regular one is about 90°.Comparing Figure S10c (substrate cleaned with piranha solution) with Figure 4c (substrate cleaned with acetone and dried using high pressure air), the terraced morphology was observed under both conditions.

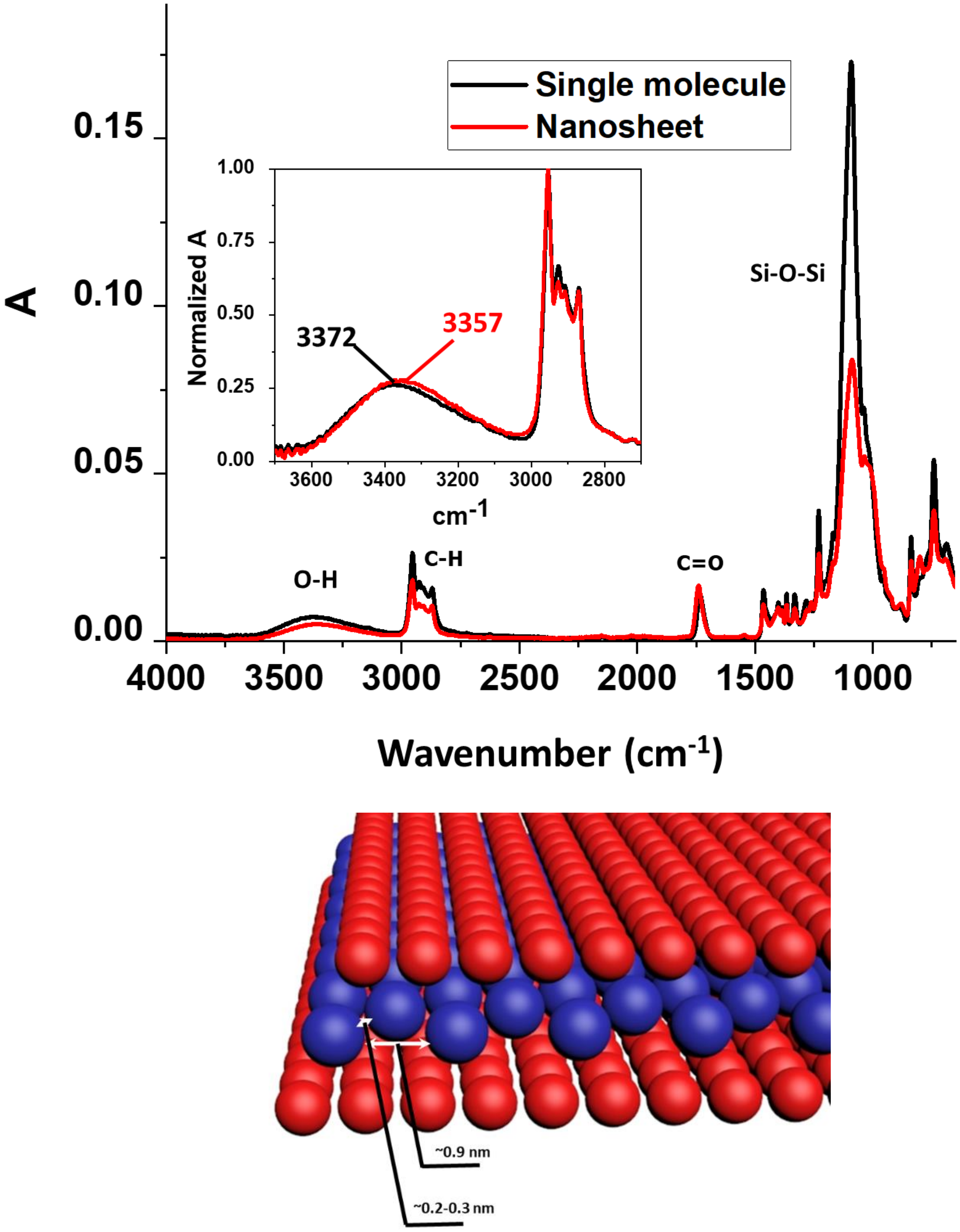

Figure S11. Up: ATR-FTIR spectra of DPOSS-BPOSS$_4$ in single molecule state (black curve) and crystallized nanosheet state (red curve). Down: An estimation of distances between DPOSS cages in the middle layer. When the macromolecule crystallized into the nanosheets, the absorbance at 3372 cm^{-1} shifted to lower wavenumber, indicating stronger H-bonding between intermolecules in the nanosheets.

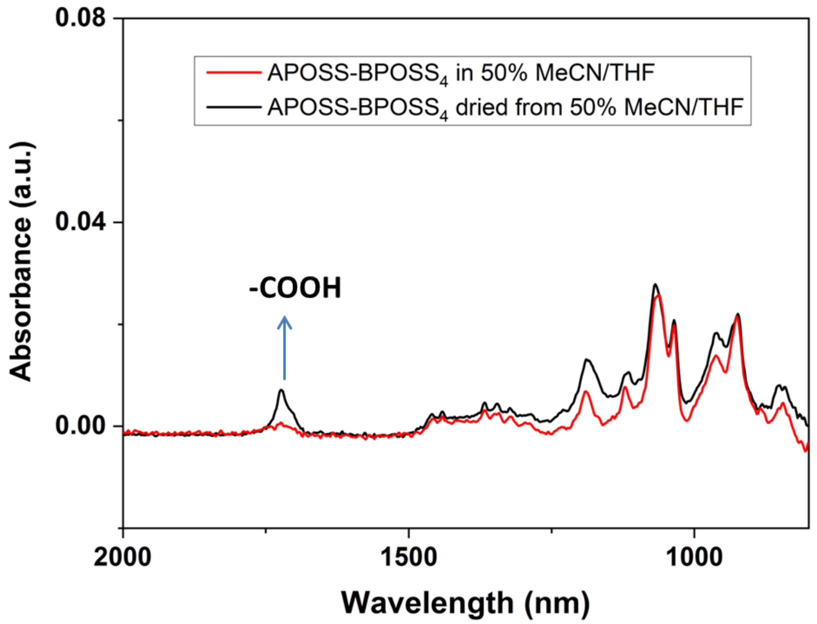

Figure S12. ATR-FTIR spectra of APOSS-BPOSS$_4$ in 50 v/v% MeCN/THF solution (red curve) and dry state (black curve). The absorbance at 1720 cm^{-1} is attributed to the C=O stretching of –COOH groups. The asymmetric stretching band of the ionized carboxylate (COO^-) groups around 1580-1620 cm^{-1} was missing, indicating the carboxyl groups were protonated in MeCN/THF.[7]

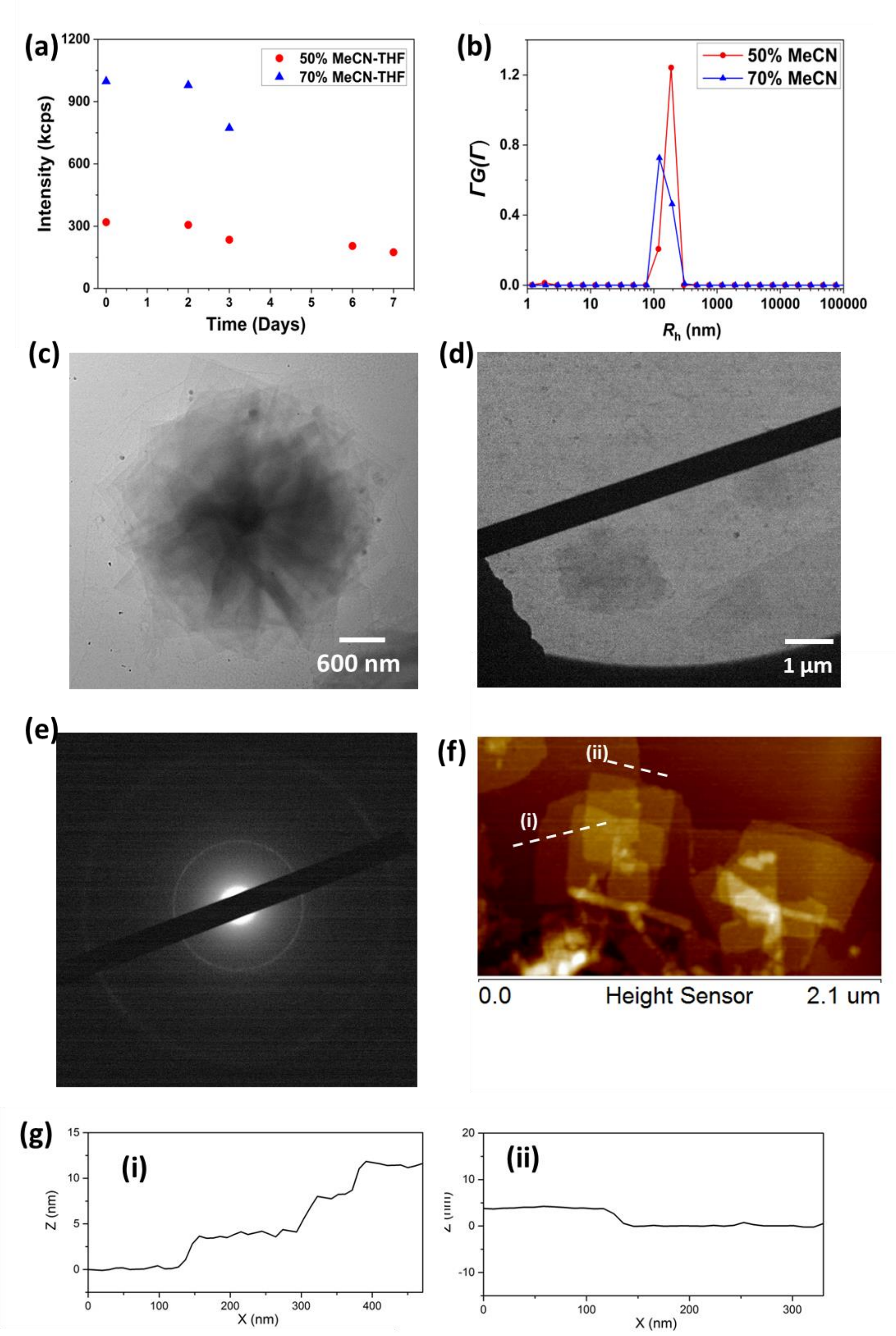

Figure S13. (a) Scattered light intensity and (b) R_h distributions of APOSS-BPOSS$_6$ in THF/MeCN mixed solvents. The R_h distributions in (b) are R_h = 164.0 nm and 135.9 nm for 50 v/v% MeCN/THF and 70 v/v% MeCN/THF, respectively. (c-d) TEM iamges, SAED pattern of (d), and (f) AFM images of APOSS-BPOSS$_6$ obtained from 50 v/v% MeCN/THF. (g) The height profile along the dash lines in (f).

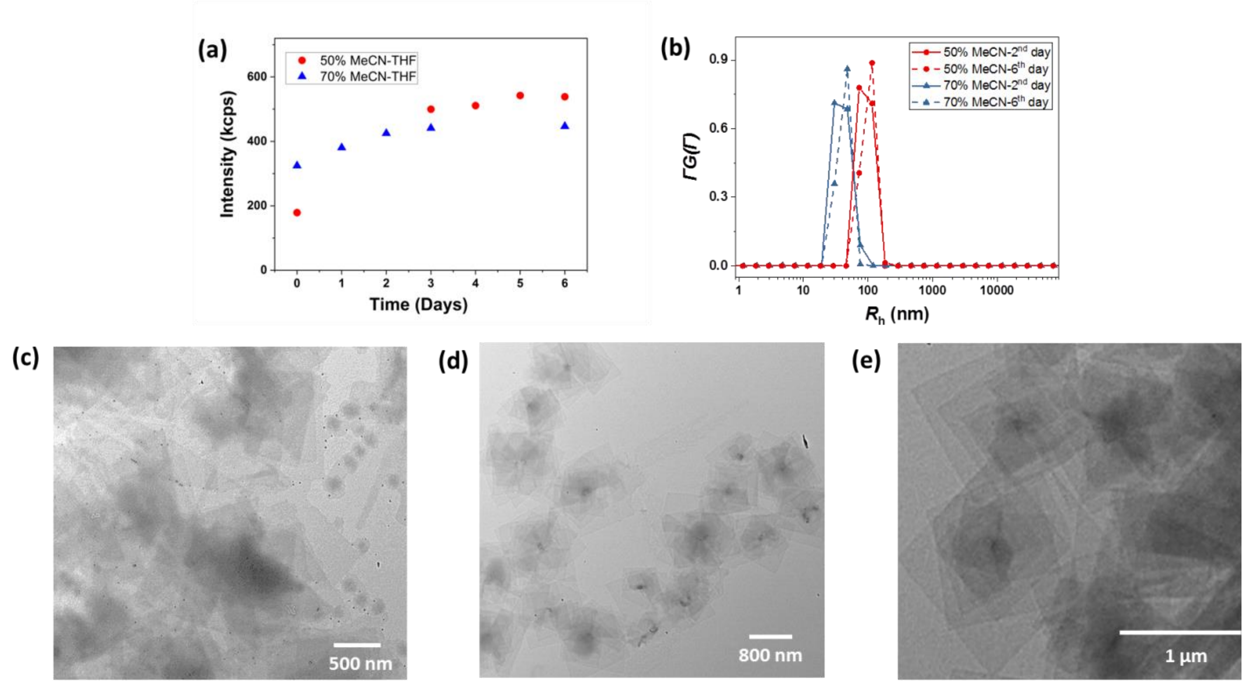

Figure S14. (a) Scattered light intensity, (b) R_h distributions of APOSS-BPOSS$_4$ in THF/MeCN mixed solvents, and (c) corresponding TEM images through fast nucleation. The R_h distributions are R_h = 92.8 nm and 45.9 nm for 50 v/v% MeCN/THF and 70 v/v% MeCN/THF, respectively. The conditions are (c) 50 v/v% MeCN/THF and (d-e)70 v/v% MeCN/THF.

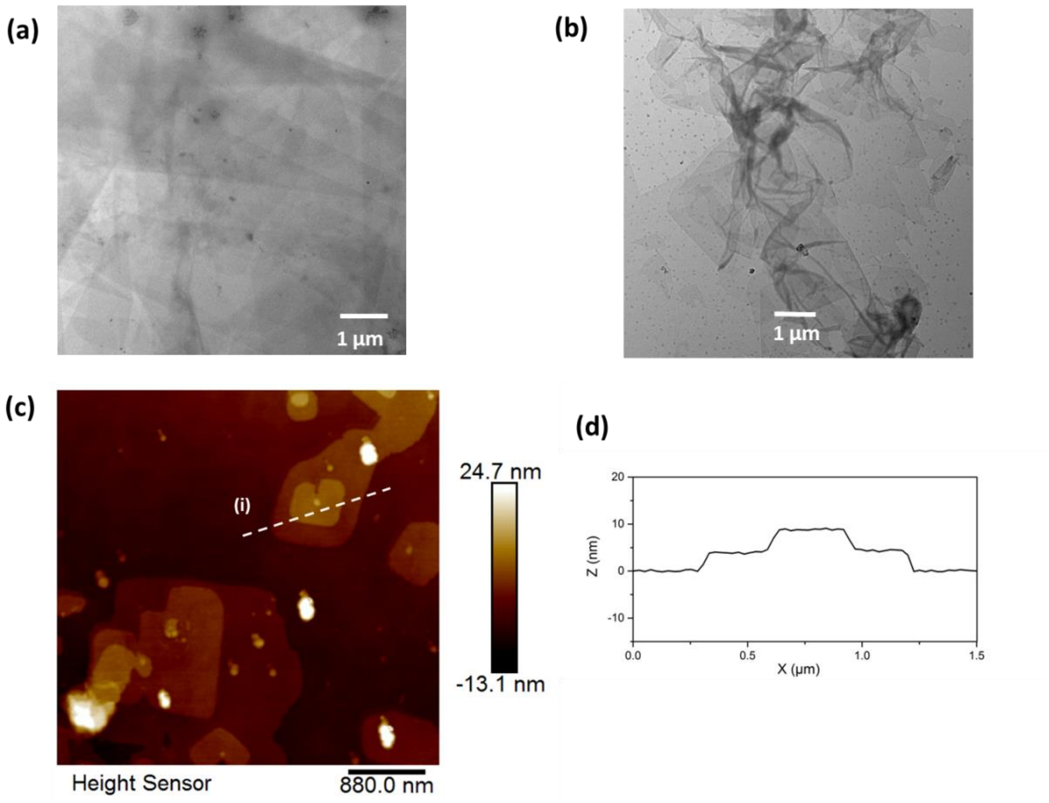

Figure S15. (a-b) TEM images and (c) AFM image of APOSS-BPOSS$_4$ obtained from (a,c) 20 v/v% and (b) 30 v/v% H_2O/dioxane mixed solvents. (d) The height profile along the dash line in (c). The thickness of the single layer was 4.1 ± 0.2 nm in average.

References:

(1) Liu, G.; Feng, X.; Lang, K.; Zhang, R.; Guo, D.; Yang, S.; Cheng, S. Z. D. Dynamics of Shape-Persistent Giant Molecules: Zimm-like Melt, Elastic Plateau, and Cooperative Glass-Like. *Macromolecules* **2017**, *50* (17), 6637–6646.

(2) Hu, J.; Peng, K.; Guo, J.; Shan, D.; Kim, G. B.; Li, Q.; Gerhard, E.; Zhu, L.; Tu, W.; Lv, W. Click Cross-Linking-Improved Waterborne Polymers for Environment-Friendly Coatings and Adhesives. *ACS Appl. Mater. Interfaces* **2016**, *8* (27), 17499–17510.

(3) Feng, X.; Zhang, R.; Li, Y.; Hong, Y. L.; Guo, D.; Lang, K.; Wu, K. Y.; Huang, M.; Mao, J.; Wesdemiotis, C.; et al. Hierarchical Self-Organization of ABnDendron-like Molecules into a Supramolecular Lattice Sequence. *ACS Cent. Sci.* **2017**, *3* (8), 860–867. https://doi.org/10.1021/acscentsci.7b00188.

(4) Feng, X.; Liu, G.; Guo, D.; Lang, K.; Zhang, R.; Huang, J.; Su, Z.; Li, Y.; Huang, M.; Li, T.; et al. Transition Kinetics of Self-Assembled Supramolecular Dodecagonal Quasicrystal and Frank–Kasper σ Phases in ABn Dendron-Like Giant Molecules. **2019**. https://doi.org/10.1021/acsmacrolett.9b00287.

(5) Yan, X.; Guo, Q.; Lin, Z.; Liu, X.; Yuan, J.; Wang, J.; Wang, H.; Liu, Y.; Su, Z.; Liu, T. Geometry-Directed Self-Assembly of Polymeric Molecular Frameworks. *Angew. Chemie* **2021**, *133* (4), 2052–2057.

(6) Shahidzadeh, N.; Schut, M. F. L.; Desarnaud, J.; Prat, M.; Bonn, D. Salt Stains from Evaporating Droplets. *Sci. Rep.* **2015**, *5*, 10335.

(7) Yu, X.; Zhong, S.; Li, X.; Tu, Y.; Yang, S.; Van Horn, R. M.; Ni, C.; Pochan, D. J.; Quirk, R. P.; Wesdemiotis, C.; et al. A Giant Surfactant of Polystyrene-(Carboxylic Acid-Functionalized Polyhedral Oligomeric Silsesquioxane) Amphiphile with Highly Stretched Polystyrene Tails in Micellar Assemblies. *J. Am. Chem. Soc.* **2010**, *132* (47), 16741–16744. https://doi.org/10.1021/ja1078305.
